# Identification of *Plasmodium falciparum* Translation Initiation eIF2β Subunit: Direct Interaction with Protein Phosphatase Type 1

**DOI:** 10.3389/fmicb.2016.00777

**Published:** 2016-05-26

**Authors:** Géraldine Tellier, Astrid Lenne, Katia Cailliau-Maggio, Alejandro Cabezas-Cruz, James J. Valdés, Alain Martoriati, El M. Aliouat, Pierre Gosset, Baptiste Delaire, Aline Fréville, Christine Pierrot, Jamal Khalife

**Affiliations:** ^1^Centre National de la Recherche Scientifique, Institut National de la Santé et de la Recherche Médicale, CHU Lille, Institut Pasteur de Lille, U1019 - UMR 8204 - Centre d'Infection et d'Immunité de Lille, Université de LilleLille, France; ^2^Centre National de la Recherche Scientifique, UMR 8576 - Unité de Glycobiologie Structurale et Fonctionnelle, Université de LilleLille, France; ^3^Institute of Parasitology, The Czech Academy of SciencesČeské Budějovice, Czech Republic; ^4^Department of Virology, Veterinary Research InstituteBrno, Czech Republic; ^5^Service d'Anatomie et de Cytologie Pathologiques, Groupe Hospitalier de l'Université Catholique de LilleLille, France

**Keywords:** *Plasmodium falciparum*, Protein Phosphatase type 1, eIF2β, protein-protein interaction, translation complex

## Abstract

Protein phosphatase 1 (PP1c) is one of the main phosphatases whose function is shaped by many regulators to confer a specific location and a selective function for this enzyme. Here, we report that eukaryotic initiation factor 2β of *Plasmodium falciparum* (PfeIF2β) is an interactor of PfPP1c. Sequence analysis of PfeIF2β revealed a deletion of 111 amino acids when compared to its human counterpart and the presence of two potential binding motifs to PfPP1 (^29^FGEKKK^34^, ^103^KVAW^106^). As expected, we showed that PfeIF2β binds PfeIF2γ and PfeIF5, confirming its canonical interaction with partners of the translation complex. Studies of the PfeIF2β-PfPP1 interaction using wild-type, single and double mutated versions of PfeIF2β revealed that both binding motifs are critical. We next showed that PfeIF2β is able to induce Germinal Vesicle Break Down (GVBD) when expressed in *Xenopus* oocytes, an indicator of its capacity to regulate PP1. Only combined mutations of both binding motifs abolished the interaction with PP1 and the induction of GVBD. In *P. falciparum*, although the locus is accessible for genetic manipulation, PfeIF2β seems to play an essential role in intraerythrocytic cycle as no viable knockout parasites were detectable. Interestingly, as for PfPP1, the subcellular fractionation of *P. falciparum* localized PfeIF2β in cytoplasm and nuclear extracts, suggesting a potential effect on PfPP1 in both compartments and raising the question of a non-canonical function of PfeIf2β in the nucleus. Hence, the role played by PfeIF2β in blood stage parasites could occur at multiple levels involving the binding to proteins of the translational complex and to PfPP1.

## Introduction

Malaria, mainly caused by *Plasmodium falciparum*, is one of the major parasitic diseases and a leading cause of morbidity and mortality throughout the tropics and sub-tropics (Burchard, 2014). Although immuno-epidemiological analyses from field studies suggest that the development of a vaccine is an achievable goal, it still faces difficulties in order to obtain high efficiency and sustainable protection (Neafsey et al., 2015; Partnership, 2015; Richie et al., 2015). These observations underline the need for continuing efforts to develop novel antimalarial drugs. The fact that the malaria parasite profoundly relies on phosphorylation/dephosphorylation post-translational modifications has meant that its kinases and phosphatases have been identified as key drug targets (Tewari et al., 2010; Solyakov et al., 2011; Doerig and Grevelding, 2015; Guttery et al., 2015). Moreover, in this context, recent reverse genetic studies on the *Plasmodium* phosphatome showed that 21 phosphatases out of 67 seem to be essential for parasite survival, including Protein Phosphatase type 1 (PfPP1) (Guttery et al., 2014).

PP1 is one of the major and most studied Ser/Thr phosphatases as it dephosphorylates a large number of proteins in different species. Functional studies show that it is a much more discriminating enzyme than previously considered (Bhattacharyya et al., 2002; Fardilha et al., 2010). PP1 is a holoenzyme composed of a highly conserved catalytic subunit (PP1c) in association with one or several regulatory subunits. The latter target PP1c to specific substrates involved in essential cellular functions such as cell division control and apoptosis (Bollen, 2001; Ceulemans and Bollen, 2004; Fardilha et al., 2010). Regulators of PP1c have been shown to be able to direct its localization and to shape its activity/specificity in a spatiotemporal manner (Hendrickx et al., 2009; Bollen et al., 2010). In mammalian cells, it has been shown that the wide regulatory network of PP1 includes nuclear and cytoplasmic regulators which control PP1c activity negatively or positively and prevent the accumulation of free PP1c, which is suggested to be toxic (Gallego and Virshup, 2005). So far, about 200 PP1 partners/regulators have been described (Heroes et al., 2013). The functional studies of diverse regulators such as SDS22, Inhibitor-2, Inhibitor-3, NIPP1, PNUTS, DARPP-32, or MYPT1 (Aggen et al., 2000; Heroes et al., 2013) considerably contributed to explaining the capacity of PP1c to participate in a multitude of cellular functions. Interestingly, human eIF2β, a known member of the eIF2 complex that controls protein synthesis, has been shown to bind PP1 (Wakula et al., 2006). This interaction was confirmed both *in vitro* and in cell lysates. Furthermore, the reported data suggest that eIF2β belongs rather to the regulator/substrate family since binding to PP1 activates the dephosphorylation of eIF2β but inhibits PP1 activity toward other substrates (Wakula et al., 2006). Structural and functional studies revealed that eIF2β contains three domains. The N-terminal domain involved in the interaction with eIF5 and eIF2B (Das et al., 1997; Das and Maitra, 2000); the central domain which is responsible for eIF2γ binding (Thompson et al., 2000) and C-terminal domain includes a region which participates in mRNA binding (Laurino et al., 1999).

In *P. falciparum*, our previous studies showed that the control of the activity of PP1 is mediated by different conserved regulators including PfLRR1 (a homolog of yeast SDS22) (Daher et al., 2006, 2007a,b), Inhibitor-2 (PfI2) (Fréville et al., 2013, 2014) and Inhibitor-3 (PfI3) (Fréville et al., 2012) with substantial differences compared to I2 and I3 orthologs in humans. Indeed, PfI2 exhibits an inhibitory role on PfPP1 activity, a canonical RVxF binding motif not present in human I2 and a peptide sequence 30% shorter than its ortholog. PfI3, although it contains the RVxF consensus motif, does not seem to be an inhibitor but rather an activator of PfPP1 *in vitro* and is unable to complement I3 deficient yeast. Whatever the regulatory role played by PfI2 or PfI3 on PP1 activity, reverse genetic analyses suggest that they are essential for *P. falciparum* growth (Fréville et al., 2012, 2013).

Taken together, these observations emphasize the importance of PP1 regulators and support the further exploration of the regulatory network of PfPP1. In this context, analyses of the *Plasmodium* genome revealed the presence of a putative *eif2*β gene (PF3D7_1010600), on chromosome 10 that could be a partner of PfPP1. Although, the examination of the deduced amino acid sequence of PfeIF2β showed an unusually short sequence, truncated at the N-terminal end when compared to its homologs, it reveals the presence of potential binding motifs to PP1. In this work, we show that PfeIF2β interacts not only as expected with PfeIF2γ and eIF5 (partners in the translation protein complex), but is also a direct interactor of PfPP1. We further identify two binding motifs in PfeIF2β involved in the interaction with PfPP1. Structure activity studies reveal that a combined mutation of these two motifs is critical to completely inhibit the functional interaction of PfeIF2β with PP1.

## Materials and methods

### Materials

pCR2.1-TOPO, pETDuet-1, pGEX4T3, and pGADT7 plasmids were purchased from Invitrogen, Novagen, Life Sciences, and Clontech respectively. pCAM-BSD and pCAM-BSD-HA plasmids were kind gifts from Dr. C. Doerig (Monash University, Melbourne, Australia).

Monoclonal anti-HA, anti-penta His, anti-GST, anti-H3, and anti-HA peroxidase antibodies were purchased from Roche, Qiagen, Sigma-Aldrich, Millipore, and Abcam respectively. Anti-actin1 and anti-SOD1 antibodies were used as previously described (Daher et al., 2006, 2010).

### Sequence analysis

Putative eIF2β, eIF2γ, and eIF5 sequences were searched using BLASTp on sequences available in PlasmoDB databases. Protein sequences (*human* and *P. falciparum*) were aligned using the ClustalW program.

### Protein classification and phylogenetic analyses

The phylogenetic analysis of eukaryotic initiation factor eIF2β was performed using 76 amino acid sequences of eIF2β from 23 apicomplexans, 5 mammals, 1 amphibian, 8 fish, 9 plants, 8 arthropods, 12 archaea, 1 yeast (fungi), 2 amoebozoa, 1 cercozoa, 1 foraminifera, and 5 excavata. Before phylogenetic analysis, sequences were submitted to the protein families database (Pfam, Finn et al., 2014) and conserved domains (CDD, Marchler-Bauer et al., 2014) for family and domain assignment, respectively. All sequences included in further analysis belonged to the eIF5/eIF2B family (pfam01873), and contained the characteristic structural domain eIF2β (Accession: PRK03988).

The sequences were aligned with MAFFT (v7), configured for the highest accuracy (Katoh and Standley, 2013). After alignment, ambiguous regions were removed with Gblocks (v0.91b) (Castresana, 2000). The final alignment contained 125 gap free amino acid positions. All evolutionary and phylogenetic analysis were performed in the Molecular Evolutionary Genetics Analysis (MEGA, v6) software (Tamura et al., 2013). The best-fit model of sequence evolution was selected based on Bayesian Information Criterion (BIC) and Akaike Information Criterion, corrected (AICc). The JTT (Jones et al., 1992) model with a proportion of Gamma distributed (G) and invariant (I) sites which had the lowest values of BIC and AICc was chosen as best-fitting model for the actual data. The evolutionary history was inferred using the Maximum Likelihood method based on JTT model and assuming a proportion of Gamma distributed (with shape parameter α = 1.19) and invariant sites (= 0.02). Initial trees for the heuristic search (Nearest-Neighbor-Interchange: NNI) were obtained by applying Neighbor-Joining algorithms and the topology with higher log likelihood value was selected. The reliability of the internal branches was assessed using the Bootstrap methods with 1000 replicates. Graphical representation and editing of the phylogenetic trees were performed with MEGA. The accession numbers of the sequences are provided in the phylogenetic tree (**Figure 2** and Table S2).

### Protein modeling

The Robetta server (http://robetta.bakerlab.org/) was used for tertiary modeling. The best structure was ranked using the RESPROX (Berjanskii et al., 2012) qualifying server. Finally, the structure was prepared using the Schrodinger's Maestro (Schrödinger: Schrödinger maestro Package In: maestro, version 99. New York: LLC; 2014) package Protein Preparation Wizard. The Protein Databank (PDB) crystal structure of *Pyrococcus furiosus* (PDB: 2DCU, chain B) was also prepared and used for a structural alignment of the two initiation factors (implemented in Maestro). *P. furiosus* (PDB: 2DCU, chain B) was chosen since this was the closest structural homolog to *P. falciparum* eIF2β according to the Dali server (Holm and Rosenström, 2010).

### Preparation of parasites

*P. falciparum* 3D7 clone was grown according to Trager et al. (Trager and Jensen, 1976), with slight modification (Fréville et al., 2012). Parasites were synchronized by a double sorbitol treatment as previously described (Vernes et al., 1984).

To isolate total DNA or proteins, parasitized erythrocytes were lysed by saponin (Umlas and Fallon, 1971) and pelleted. Soluble proteins extracts were prepared according to Fréville et al. (2013).

Genomic DNA (gDNA) was extracted using the KAPA Express Extract kit (KAPABioSystem) as described in the manufacturer's protocol.

### Recombinant protein expression and purification

The coding regions of PfeIF2β and PfeIF2γ were obtained by RT-PCR using the primers described in Table S1 and subcloned in *E. coli* expression vectors pETDuet-1 and pGEX4T3 which allows the expression of proteins fused with a 6-His- or GST-tag respectively. For the expression of PfeIF5 or PfeIF2β in *Xenopus* oocytes, they were amplified with p12–p13 and p14–p15 respectively (Table S1) and subcloned in pGADT7 vector allowing the production of capped RNA (cRNA) using the T7 promoter. cRNA was obtained as previously described (Fréville et al., 2014) and used for the expression of HA tagged proteins in oocytes.

For mutant constructs of PfeIF2β, PCR-based site-directed mutagenesis (Qbiogene) was used. The pETDuet-PfeIF2β plasmid was used as template with primers p4–p5 and p6–p7 (Table S1) to obtain PfeIF2β-^29^AGEAKA^34^ and PfeIF2β-^103^KAAA^106^ respectively. For the PfeIF2β-^29^AGEAKA^34^/^103^KAAA^106^ mutant construct, it was obtained by PCR using the primers p6–p7 and the PfeIF2β-^29^AGEAKA^34^ plasmid as template. All the open reading frames (ORFs) as well as the mutation points were checked by sequencing.

### Recombinant protein expression and antisera production

Expression and purification of PfPP1 was previously described (Fréville et al., 2013). The expression of wild PfeIF2β recombinant protein and the mutated versions were carried out in the *E. coli* BL21 strain as previously described (Fréville et al., 2013) with slight modifications. Briefly, inductions were carried out at 30°C for 3 h in the presence of 50 μM ZnCl_2_ and tagged-proteins purifications were done under non-denaturing conditions as described by manufacturers' protocol using Ni-NTA agarose beads (Qiagen). The purity, checked by 15% SDS-PAGE followed by SimplyBlue™ safe staining (Invitrogen), was >90%. Recombinant PfeIF2β-6His protein was subjeicted to peptide mass fingerprint by MALDI-TOF mass spectrometry to confirm its identity.

For the GST tagged proteins, the expression was induced at 37°C for 3 h in the presence of 0.5 mM IPTG and 1 mM MnCl2 for PfPP1-GST or 25 mM MgCl2 and 50 μM ZnCl2 for the expression of PfeIf2β-GST and PfeIf2γ-GST. Proteins were purified according to the manufacturers' instructions using glutathione Sepharose beads (Sigma). For GST pull down experiments, purified recombinant proteins were bound to glutathione-Sepharose beads overnight at 4°C and washed with a buffer containing 20 mM Tris-HCl pH7.4, 500 mM NaCl, 50 μM ZnCl_2,_ and 0.1% Triton X-100 before use.

The antisera anti-PfeIF2β was raised according to the protocol previously described (Fréville et al., 2013).

### GST pull-down assays

Two μg of PfeIF2β-6His recombinant protein (wild-type or mutated) were incubated with PfPP1-GST, PfeIF2γ-GST or GST bound to gluthatione-Sepharose beads, and 25 μg of BSA in binding buffer (20 mM Tris-HCl pH7.4, 500 mM NaCl, 20 mM Hepes, 0.2 mM EDTA, 0.1% Triton X-100, 1 mM DTT, protease cocktail inhibitor and 1 mM MnCl_2_, 50 μM ZnCl_2_ or 25 mM MgCl_2_ according to uses proteins) for 1–2 h at 4°C on wheel. After five washes with binding buffer, proteins were eluted in loading buffer, separated on SDS-PAGE and blotted to nitrocellulose. Blots were revealed with anti-His or anti-GST mAb antibodies. Horseradish peroxidase labeled anti-mouse (1:50,000) was used as secondary antibodies followed by chemiluminescence detection (DURA, Pierce).

### Induction of xenopus oocytes GVBD and co-immunoprecipitation experiments

Preparation of *Xenopus* oocytes and microinjection experiments were performed as previously described (Vicogne et al., 2004). Briefly, in each assay, 20 oocytes removed from at least two or three different animals were microinjected with PfeIF2β-6His (wild-type or mutated) recombinant protein. Progesterone was used as a positive control for oocyte maturation. GVBD was detected by the appearance of a white spot at the apex of the animal pole after 15 h. In order to carry out immunoprecipitation, extracts from 20 oocytes removed from at least two or three animals were prepared 15 min after the microinjection of PfeIF2β (wild-type or mutated) as previously described (Vandomme et al., 2014).

Xenopus oocytes were also used in order to test the interaction of PfeIF2β with its partners PfeIF2γ and PfeIF5. cRNA corresponding to HA-PfeIF2β or HA-PfeIF5 proteins was microinjected followed by the microinjection of His-PfeIF2γ or His-PfeIF2β recombinant proteins respectively. Protein extractions, immunoprecipitation and immunoblots experiments were carried out as previously described (Vandomme et al., 2014).

To examine the interaction of PfeIF2β with XePP1, the experiments were performed as previously described (Fréville et al., 2013).

### Detection of PfeIF2β in *P. falciparum*

For western blots, 10 μg/ lane of *P. falciparum* soluble proteins from asynchronous cultures was separated on a 4–20% SDS-PAGE and blotted onto nitrocellulose. For the detection of PfeIF2β, blots were probed with primary mouse anti-PfeIF2β serum (1:1000 in PBS milk 5%).

The detection of native PfeIF2β in total proteins extracted from asynchronous cultures of parasites was carried out by using PfPP1-6His beads. After pre-clearing on Ni-NTA agarose beads, 2 mg of total protein extracts were incubated at 4°C overnight with PfPP1-6His beads. After washing steps, proteins were eluted with SDS-PAGE loading buffer, separated by SDS-PAGE and blotted to nitrocellulose. Blots were revealed with pre-immune serum, anti-PfeIF2β serum, or anti-His mAb antibodies. Horseradish peroxidase labeled anti-mouse (1:50,000) was used as secondary antibodies followed by chemiluminescence detection (DURA, Pierce).

### Generation of *P. falciparum* transgenic parasites

The *PfeIF2*β disruption plasmid (pCAM-*PfeIF2*β) was generated by the insertion of a PCR product corresponding to a 5′ portion from the PfeIF2β sequence (846 bp) into the pCAM-BSD vector which contains a cassette conferring resistance to blasticidin. The insert was obtained using 3D7 genomic DNA as template and the primers p18–p19 (with PstI and BamHI sites respectively, Table S1). Attempts to check the accessibility of the *PfeIF2*β locus were performed by transfecting wild 3D7 parasites with 3′ tagging constructs. To this end, the 3′ end of the *PfeIF2*β sequence (845 bp, omitting the stop codon) was amplified by PCR using 3D7 genomic DNA and the primers p16–p17 (containing PstI and BamHI restriction sites respectively, Table S1). The 3′ tagging plasmids were generated by inserting the PCR product into the pCAM-BSD-hemagglutinin (HA). Ring stage 3D7 parasites were transfected with 100 μg of plasmid DNA by electroporation, according to Sidhu et al. (2005). To select transformed parasites, 48 h after transfection, blasticidin antibiotic (Invivogen) was added to a final concentration 2.5 μg/ml. Resistant parasites appeared after 4–6 weeks and were maintained under drug selection.

### Genotype and phenotype analysis of *P. falciparum* transfectants

To verify the presence of correct constructs in transfected parasites, plasmid rescue experiments were carried out. Genomic DNA extracts (KAPA Express Extract) from wild-type or transfected parasites were used to transform DH5α *E. coli* cells (Invitrogen). Plasmid DNA was purified from bacterial clones and digested with restriction enzymes (PstI and BamHI).

Genotypes of *Pfeif2*β Knock-Out parasites were analyzed by PCR on genomic DNA using the primers p24 (derived from the 5′ non-translated region and absent in the construct) and p30 (Table S1) specific for the pCAM-BSD vector. Genotypes of *Pfeif2*β Knock-In parasites were analyzed using primers p24–p31 (reverse primer corresponding to HA tag, Table S1).

### Immunolabeling assays

Five milliliters of unsynchronized blood cultures of parasites *P. falciparum* 3D7 or PfeIF2β-HA recombinant strain (5% parasitemia) were centrifuged (700 g, 5 min), and the pellet was fixed 24 h with 10% neutral formalin and paraffin embedded. Morphological assessment was obtained by examining sections (4 μm) stained with hematoxylin-eosin-safran.

Immunofluorescence assays (IFA) were done to immunolocalize PfeIF2β-HA tag recombinant proteins. Sections of *P. falciparum* eIF2β-HA tag recombinant strain were incubated for 1 h at 37°C with an anti-HA tag (biotine) rabbit polyclonal antibody (1:100 dilution; Abcam). After washing in PBS, sections were incubated with streptavidine-Alexa fluor 488-labeled (1:200; Invitrogen) added with 4′,6-diamidino-2-phenylindole dihydrochloride (DAPI, 0.2 μg/ml; Invitrogen) for 1 h at 37°C. After washing, slides were mounted with an anti-fade mounting medium (Bio-Rad) and analyzed using a Zeiss LSM880 confocal microscope (Zeiss).

### Subcellular fractionation

The cytoplasmic and nuclear extracts were prepared as previously described (Voss et al., 2002). Sorbitol synchronized parasites at ring-stage (10–15 hpi), trophozoite-stage (22–28 hpi), and schizont-stage (40–42 hpi) were incubated in lysis buffer containing 20 mM HEPES pH7.8, 10 mM KCl, 1 mM EDTA, 1 mM DTT, 1% Triton X-100, and protease inhibitor cocktail for 5 min on ice. After centrifugation at 2500 g, the supernatants corresponding to the cytoplasmic fractions were recovered. To extract the nuclei, the pellets were washed twice with the lysis buffer and were further incubated in extraction buffer containing 20 mM HEPES pH7.8, 800 mM KCl, 1 mM EDTA, 1 mM DTT, and protease inhibitor at 4°C on a rotator for 30 min. The nuclear extracts were centrifuged at 12,000 g for 30 min and the supernatants corresponding to the nuclear extracts were harvested. Ten micrograms were used in Western blot assays as described above. The band intensities were quantified using the Image quant TL8.1 software (GE Healthcare, Imager Las 4000) in which the band intensity of ring nuclear fraction. The results represent the mean of fold change between nuclear and cytoplasm fractions.

## Results

### Sequence analysis of eIF2β and its expression by *P. falciparum*

**Figure 1:**
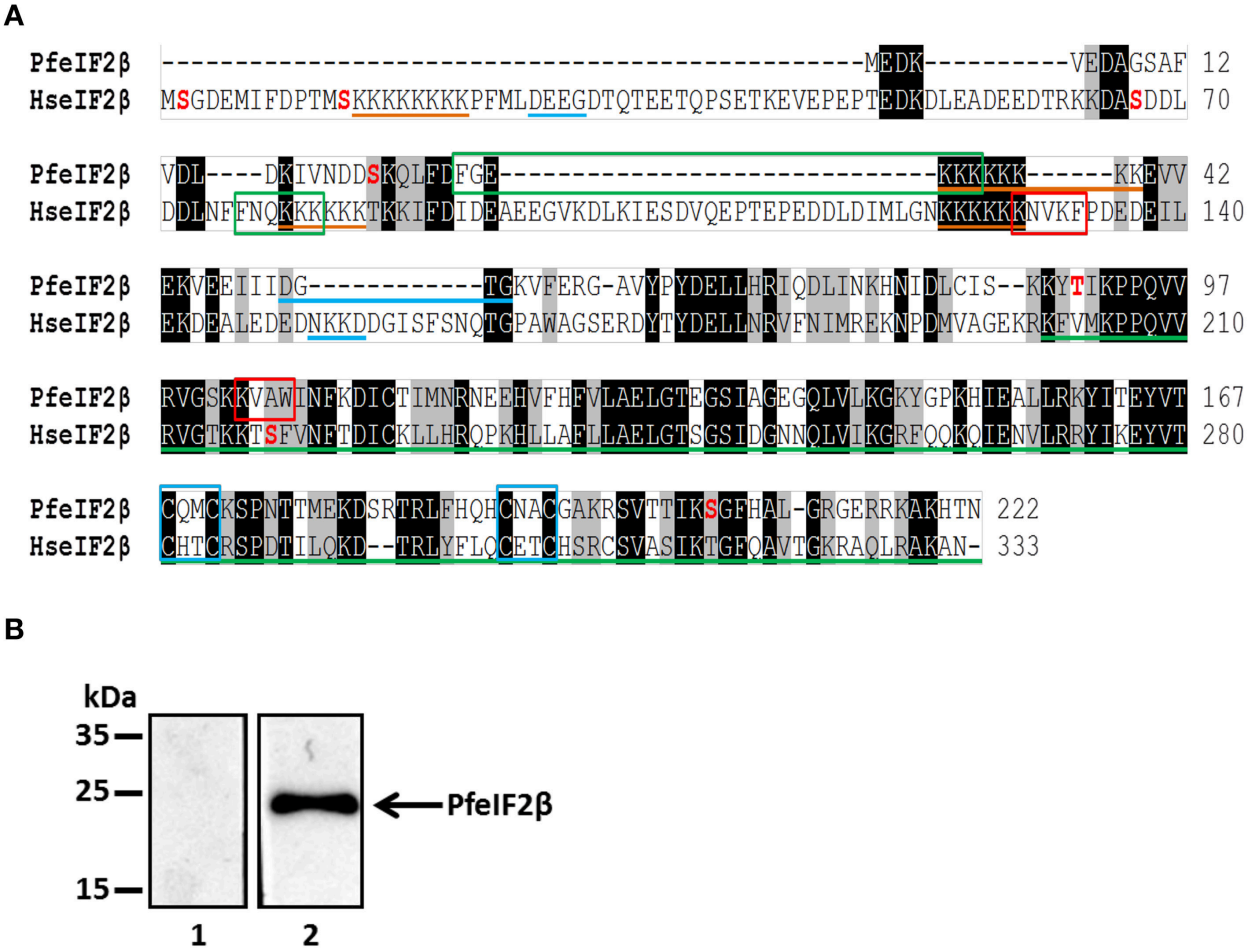
**Molecular cloning, sequences analysis of PfeIF2β and its expression by ***P. falciparum.***** **(A)** Analysis of *P. falciparum* (PF3D7_1010600) and human eIF2β (GenBank AAA52383.1). Sequences were aligned using ClustalW Multiple Alignment (BioEdit). The identical and semiconserved amino acids are highlighted in black and gray respectively. Lysine blocks and GTP binding domains are underlined with orange and blue lines respectively. Green line corresponds to the domain of the superfamily eIF2/eIF5. The blue boxes contain the ≪zinc finger≫ motif (C_2_-C_2_motif). The potential PP1-binding motifs “RVxF” and “FxxR/KxR/K” are in red and green boxes respectively. Phosphorylable amino acids reported in PlasmoDB are in red. **(B)** Detection of endogenous PfeIF2β in asynchronous cultures of *P. falciparum*. Blots were probed with pre-immune serum (lane 1) or with anti-PfeIF2β serum (lane 2). The blots were revealed as described in Section Materials and Methods.

The eIF2β gene bioinformatically identified in the *P. falciparum* genome (PfeIF2β) (accession number PF3D7_1010600) encodes a sequence of 222 amino acids, which is 111 amino acids shorter than human eIF2β. To clearly identify the ORF of this gene, PCRs using internal primers of the coding sequence and primers (Table S1) derived from genomic DNA were performed on cDNA obtained from total RNA. This approach determined the start and stop codons with an ORF of 666 nucleotides (Figure S1). The deduced sequence of 222 amino acids, with an expected molecular mass of 25.3 kDa, showed an overall identity with human eIF2β of 47% (Figure 1A). This identity is mainly observed at the C-terminus (nearly 150 residues) which contains a conserved region also present in the eIF5 family. Closer examination of PfeIF2β, revealed the presence of one lysine block and one GTP binding motif while the human counterpart contains 3 lysine blocks, known to bind to mRNA, and 2 GTP binding motifs (Figure 1A). In addition, PfeIF2β possesses one canonical binding motif, RVxF (^103^KVAW^106^) as well as the FxxR/KxR/K (^29^FGEKKK^34^) motif, described to be involved in binding to PP1. The presence of four conserved cysteines in PfeIF2β (Figure 1A), known to be involved in the structural stability of the C-terminus in the presence of Zinc ion (Gutiérrez et al., 2002), should also be noted.

To further confirm the expression of eIF2β by *P. falciparum* at its expected size, an antiserum raised against the His-tagged recombinant protein (Figure S2) was used in immunoblots on a total extract of blood parasites from an asynchronous culture. As shown in Figure 1B (lane 2), the immunoblot showed a specific band at 25 kDa confirming the expected size of PfeIF2β and excluding a longer form of this protein. Proteomic analysis available at the PlasmoDB revealed that PfeIF2β is expressed during the intraerythrocytic development of *P. falciparum* (Pease et al., 2013).

### Phylogenetic analyses

**Figure 2:**
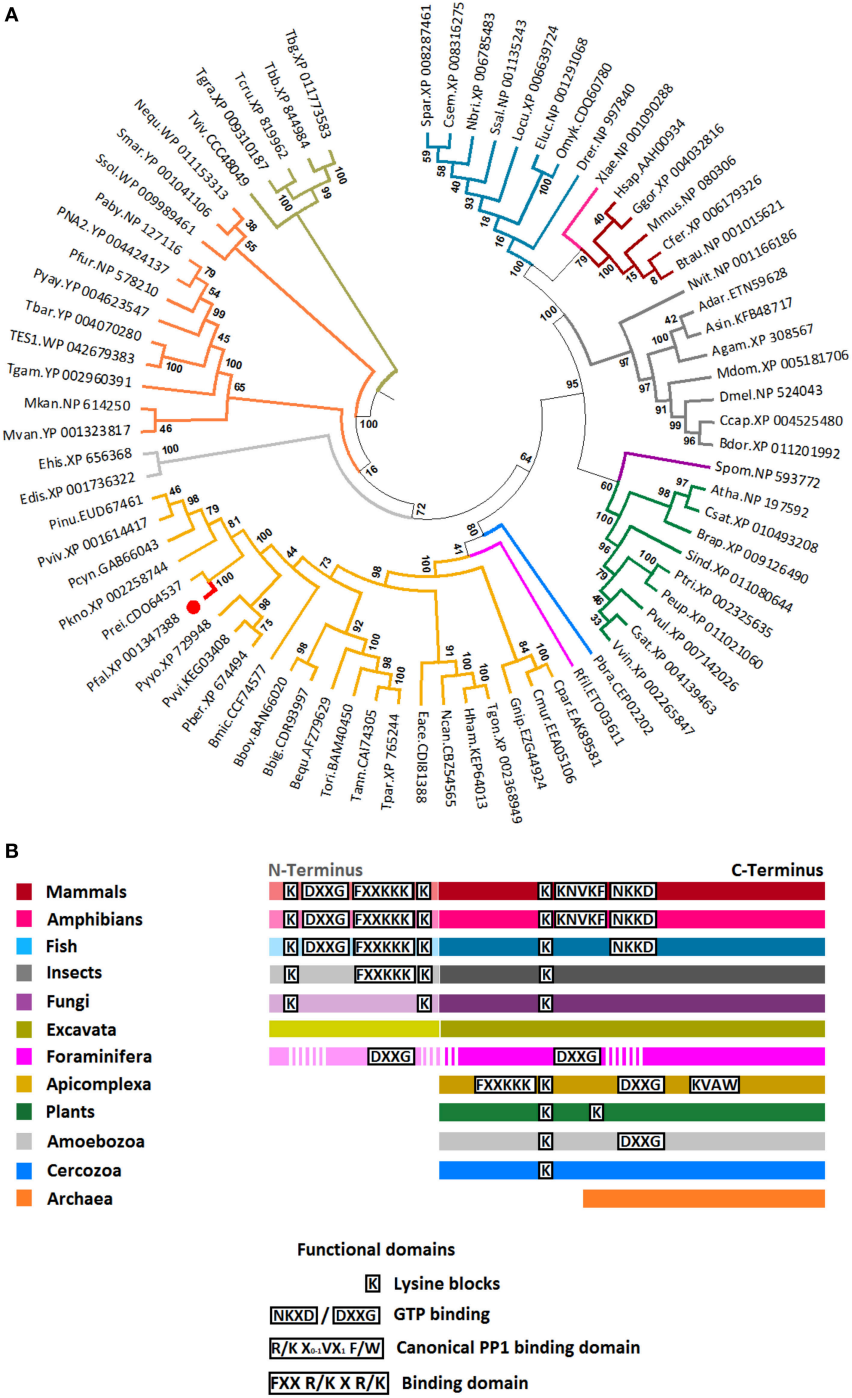
**Phylogenetic position and relevant domains of eIF2β from Apicomplexan parasites. (A)** The figure displays the maximum likelihood phylogenetic tree of eIF2β amino acid sequences from 23 apicomplexan, 5 mammals, 1 amphibian, 8 fish, 9 plants, 8 arthropods, 12 archaea, 1 yeast (fungi), 2 amoebozoa, 1 cercozoa, 1 foraminifera, and 5 excavata. All sequences selected for the analysis belonged to eIF5/eIF2B family (pfam01873), and contained the characteristic eIF2B domain (Accession number: PRK03988). The position eIF2β from *P. falciparum* is shown (red branch and circle). Numerals at internal branches represent bootstrap values. Only bootstrap values higher than 50 are shown. The accession number of each sequence is provided in the figure. The name of species was abbreviated as follow: Apicomplexan: Pfal*, Plasmodium falciparum;* Pber*, P. berghei;* Pkno*, P. knowlesi;* Pviv*, P. vivax;* Pyyo*, P. yoelii yoelii;* Prei*, P. reichenowi;* Pcyn*, P. cynomolgi;* Pinu*, P. inui;* Pvvi*, P. vinckei vinckei;* Bequ*, Babesia equi;* Bbig*, B. bigemina;* Bmic*, B. microtis;* Bbov*, B. bovis;* Tann*, Theileria annulata;* Tori*, T. orientalis;* Tpar*, T. parva;* Hham*, Hammondia hammondi;* Eace*, Eimeria acervulina;* Ncan*, Neospora caninum;* Cpar*, Cryptosporidium parvum;* Cmur*, C. muris;* Tgon, *Toxoplasma gondii*; and Gnip, *Gregarina niphandrodes*; Mammals: Hsap*, Homo sapiens;* Mmus*, Mus musculus;* Btau*, Bos taurus;* Ggor*, Gorilla gorilla;* Cfer*, Camelus ferus*; Amphibian: Xlae, *Xenopus laevis*; Fish: Drer*, Danio rerio;* Ssal*, Salmo salar;* Eluc*, Esox Lucius;* Omyk*, Oncorhynchus mykiss;* Locu*, Lepisosteus oculatus;* Spar*, Stegastes partitus;* Csem*, Cynoglossus semilaevis;* Nbri, *Neolamprologus brichardi*; Plant: Atha*, Arabidopsis thaliana;* Pvul*, Phaseolus vulgaris;* Csat*, Cucumis sativus;* Sind*, Sesamum indicum;* Csat*, Camelina sativa;* Vvin*, Vitis vinifera;* Ptri*, Populus trichocarpa;* Peu*, Populus euphratica;* Brap, *Brassica rapa*; Insects: *Drosophila melanogaster*; Ccap*, Ceratitis capitata;* Bdor*, Bactrocera dorsalis;* Adar*, Anopheles darling;* Asin*, A. sinensis;* Agam*, A. gambiae;* Mdom*, Musca domestica;* Nvit*, Nasonia vitripennis*; Archeae: Nequ*, Nanoarchaeum equitans;* Ssol*, Sulfolobus solfataricus;* PNA2, *Pyrococcus* sp. NA2; TES1, *Thermococcus* sp. ES1; Tbar*, T. barophilus;* Tgam, *T. gammatolerans* EJ3; Paby*, Pyrococcus abyssi* GE5; Pyay, *P. yayanosii* CH1; Pfur*, P. furiosus;* Mvan, *Methanococcus vannielii* SB*;* Mkan, *Methanopyrus kandleri* AV19; Smar*, Staphylothermus marinus* F1; and the Fungi: Spom*, Schizosaccharomyces pombe*; Amoebozoa: Edis*, Entamoeba dispar;* Ehis*, Entomoeba histolytica;* Cercozoa: Pbra*, Plasmodiophora brassicae;* Foraminifera: Rfil*, Reticulomyxa filose* and Excavata: Tviv*, Trypanosoma vivax;* Tgra*, T. grayi;* Tbb*, T. brucei brucei;* Tbg*, T. brucei gambiense;* Tcru, *T. cruzi*. The accession number of each sequence is provided in Table S2. **(B)** Simplified representation of eIF2β sequence for each group in the tree. Data regarding functional domains was collected from Asano et al. (1999) and Fréville et al. (2014).

All sequences included in the phylogenetic analysis belonged to the eIF-5/eIF-2B family (pfam01873), and contained the characteristic structural domain eIF-2B (Accession: PRK03988). The evolutionary history of 76 eIF2β amino acid sequences from apicomplexan, mammals, amphibian, fish, plants, arthropods, archaea, fungi, amoebozoa, cercozoa, foraminifera, and excavata was inferred using the maximum likelihood method based on the JTT+G+I model (see Section Materials and Methods). The tree topology with the highest log likelihood is shown in Figure 2A. The eIF2β of all independent taxa included in the analysis formed monophyletic clades, except for archaea that was fractioned in two clusters. The topology of tree reflects well the current classification of Eukaryotes (Adl et al., 2012). For example, the eIF2β sequences from Apicomplexan (Alveolata), Cercozoa and Foraminifera clustered together. Members of Metazoa (Opisthokonta) formed a monophyletic clade, but the fungi *Schizosaccharomyces pombe* (Opisthokonta) clustered together with Plantae (Archaeplastida) and no with the Metazoan as expected (Figure 2A). However, the molecular structure of *S. pombe* eIF2β resembles that of Metazoan and no Plantae. A remarkable difference between Opisthokonta and Archaeplastida eIF2β is that while members of Opisthokonta present an N-terminus with two lysine blocks, members of Plantae lack this domain (Figure 2B). The N-terminus is also absent in Apicomplexa, Amoebozoa, Cercozoa, and Archaea. Consequently, these groups of organisms do not present the additional two lysine blocks found in the N-terminus of Opisthokonta. It is noteworthy that although *Reticulomyxa filose* (Foraminifera) and *Trypanosoma* spp. (Excavata) present an N-terminus, it is not similar to that found in Opisthokonta (Figure 2B). From this phylogenetic tree, the most parsimonious explanation for the evolution of the N-terminal extension is that it evolved independently in Opisthokonta, Excavata, and Foraminifera probably and may have different functions within each of these groups. In agreement with this, the eIF2β from Excavata and Foraminifera do not present the typical lysine blocks found in all the other Eukaryotes. Remarkably, despite the fact that Apicomplexa eIF2β lack the N-terminus, it presents the two main PP1 binding domains found in Mammals and Amphibians (FxxR/KxR/K and R/KxV/IxF/W), which suggests a conserved function (Figure 2B).

**Figure 3:**
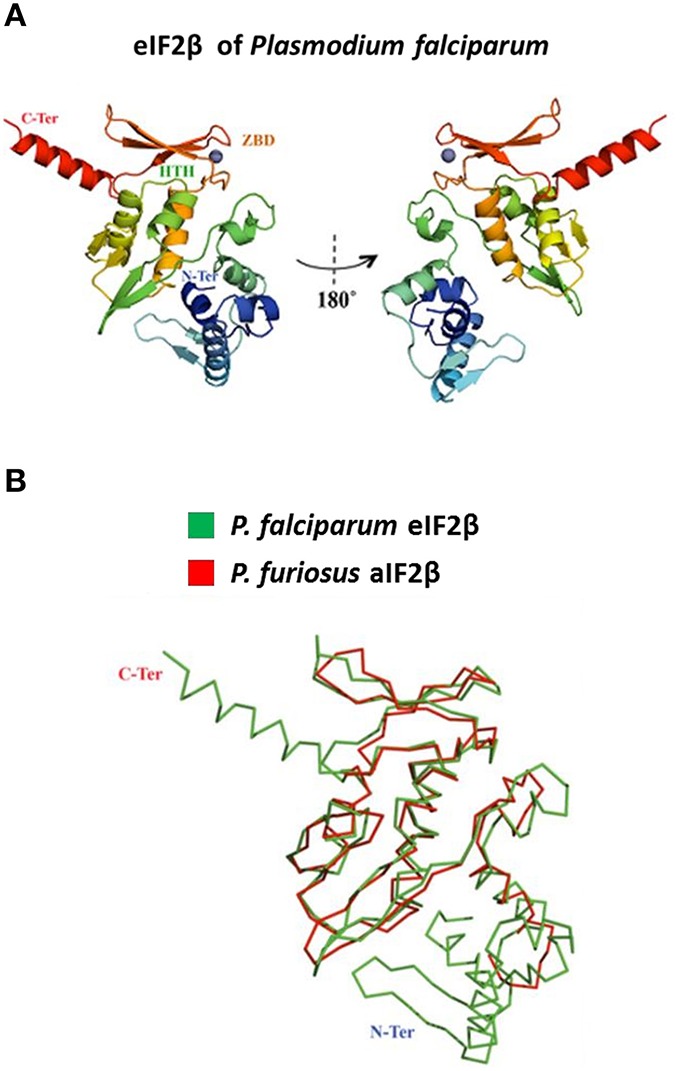
**Modeling of the tertiary structure of PfeIF2β. (A)** The figure shows the tertiary structure for the initiation factor of *P. falciparum* (predicted model) based on the initiation factor of *Pyrococcus furiosus* (PDB: 2DCU, chain B) in 180 turns. The structure is color-coded from the N-terminus (blue) to the C-terminus (red). The helix-turn-helix (HTH) and the zinc-binding domain (ZBD) are indicated (colored according to the respective position). The zinc ion is represented as a gray sphere. **(B)** The panel is a superposition of the alpha-carbon backbone of both structures (RMSD = 2.3Å).

Using the Multiple Mapping Method (http://www.fiserlab.org/servers/M4T), we observed that the putative tertiary structure of *P. falciparum* eIF2β (60–201 residues) was similar to the architecture of aIF2β of *P. furiosus* (PDB: 2DCU, chain B) with an α*ββααββαββ* topology where the last two β sheets are conserved for zinc ion binding (Figure 3A). The helix-turn-helix domain interacts with the γ-domain of initiation factor (Gutiérrez et al., 2004; Sokabe et al., 2006) and Figure 3B shows that its position and conformation are conserved. Since the entire sequence of the β-domain was used, the modeled *P. falciparum* eIF2β depicts the additional ~30 residues extending both termini.

### Interaction of PfeIF2β with PfeIF2γ and PfeIF5

**Figure 4:**
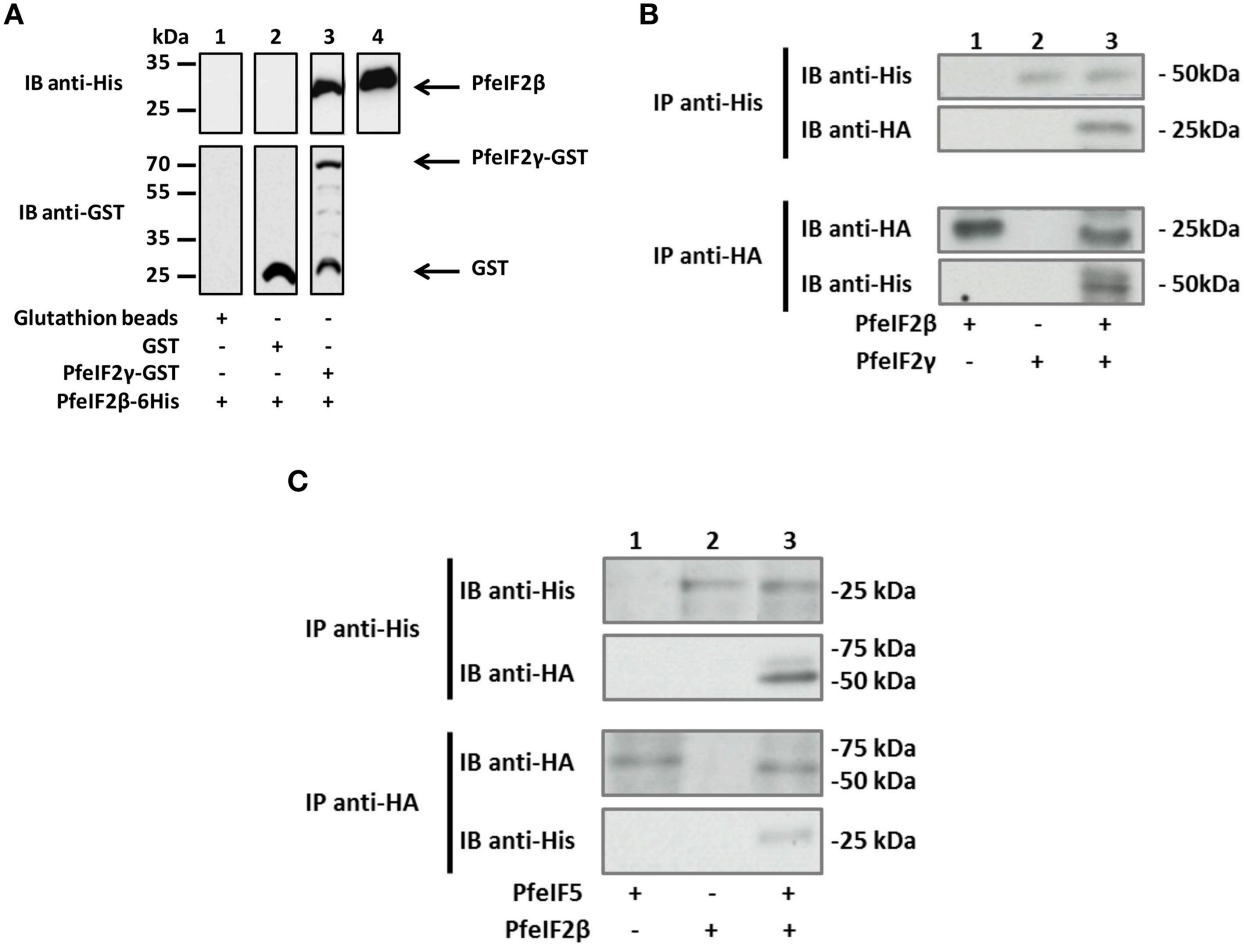
**Interaction between PfeIF2β and its partners PfeIF2γ and PfeIF5. (A)** GST pull-down assays. Glutathione beads alone (lane 1) or coupled with GST alone (lane 2), or GST-PfeIF2γ (lane 3) were incubated with 6His-tagged PfeIF2β wild-type. After washings, proteins bound to the beads were separated by SDS-PAGE and blotted to nitrocellulose. Immunoblot (IB) analysis was performed with mAb anti-His antibodies (upper blot) and mAb anti-GST (lower blot). As control, 20% of the input of PfeIF2β protein detected was used and immunoblotted with anti-His antibody (lane 4). **(B)** Interaction of PfeIF2β with PfeIF2γ in *Xenopus* oocytes. His-tagged PfeIF2γ recombinant protein and cRNA of PfeIF2β producing HA-tagged protein were micro-injected in fresh oocytes. Co-immunoprecipitations were carried out with anti-His (recognizing recombinant PfeIF2γ tagged with 6-His) (upper blot) or with anti-HA (recognizing PfeIF2β tagged HA) (lower blot) antibodies from micro-injected *Xenopus* extracts. Immunoprecipitates from *Xenopus* oocytes micro-injected with cRNA PfeIF2β alone (lane 1), PfeIF2γ protein alone (lane 2), or PfeIF2β and PfeIF2γ (lane 3) were eluted, separated by SDS-PAGE and transferred to nitrocellulose membrane. Immunoblot (IB) analysis was performed with anti-His or anti-HA antibodies. **(C)** Interaction of PfeIF2β with PfeIF5. His-tagged PfeIF2β recombinant protein and cRNA of PfeIF5 producing HA-tagged protein were micro-injected in fresh oocytes. Co-immunoprecipitations were performed as described in **(B)**. Immunoprecipitates (IP) from *Xenopus* oocytes micro-injected with PfeIF5 cRNA alone (lane 1), PfeIF2β protein alone (lane 2), or PfeIF2β and PfeIF5 cRNA (lane 3) were eluted, separated by SDS-PAGE and transferred to nitrocellulose membrane. Immunoblots were performed as described in **(B)**.

Since eIF2β is known to play a role in the initiation of translation and had been reported to be a component of the translational machinery by interacting with eIF2γ and eIF5, we next examined the ability of PfeIF2β to interact with the corresponding initiation factors present in *P. falciparum*. Both PfeIF2γ (PF3D7_1410600) and PfeIF5 (PF3D7_1206700) were obtained and sequenced. Deduced amino acid sequence analyses showed that both proteins exhibited conserved regions known to be involved in the interaction with eIF2β (Figures S3A,B). The expression in *E. coli* of recombinant tagged protein was successful for PfeIF2γ but failed for PfeIF5, which could be related to the lack of expression due to the presence of amino acid repeats and/or to toxicity for bacteria. We then tested by a pull-down assay whether PfeIF2β interacts with PfeIF2γ. Results presented in Figure 4A (lane 3) indicated that PfeIF2β was indeed able to bind GST-PfeIF2γ. The direct interaction was supported by the absence of eIF2β binding to GST alone (Figure 4A, lane 2). In an independent approach, we further confirmed this PfeIF2β-PfeIF2γ interaction using *Xenopus* oocytes model where the recombinant cRNA of PfeIF2β (expressing a HA-tagged protein) and His-tagged recombinant protein PfeIF2γ were micro-injected. Co-immunoprecipitation with either anti-His or anti-HA antibodies followed by immnunoblotting analyses clearly showed an interaction between PfeIF2β and PfeIF2γ (Figure 4B, lane 3). The use of anti-His or anti-HA antibodies on extracts from singly micro-injected oocytes (Figure 4B, lanes 1 and 2) confirmed the specificity of the interaction.

To further examine the capacity of PfeIF2β to interact with PfeIF5, the recombinant cRNA of PfeIF5 (expressing a HA-tagged protein) and His-tagged recombinant protein PfeIF2β were micro-injected into *Xenopus* oocytes. The co-immunoprecipitation/immunoblot assays clearly revealed an interaction between PfeIF2β and PfeIF5 (Figure 4C, lane 3). Taken together, these experiments indicate that the proteins produced are correctly folded and that PfeIF2β binds to proteins of the translation initiation eIF complex.

### Interaction of PfeIF2β with PfPP1

**Figure 5:**
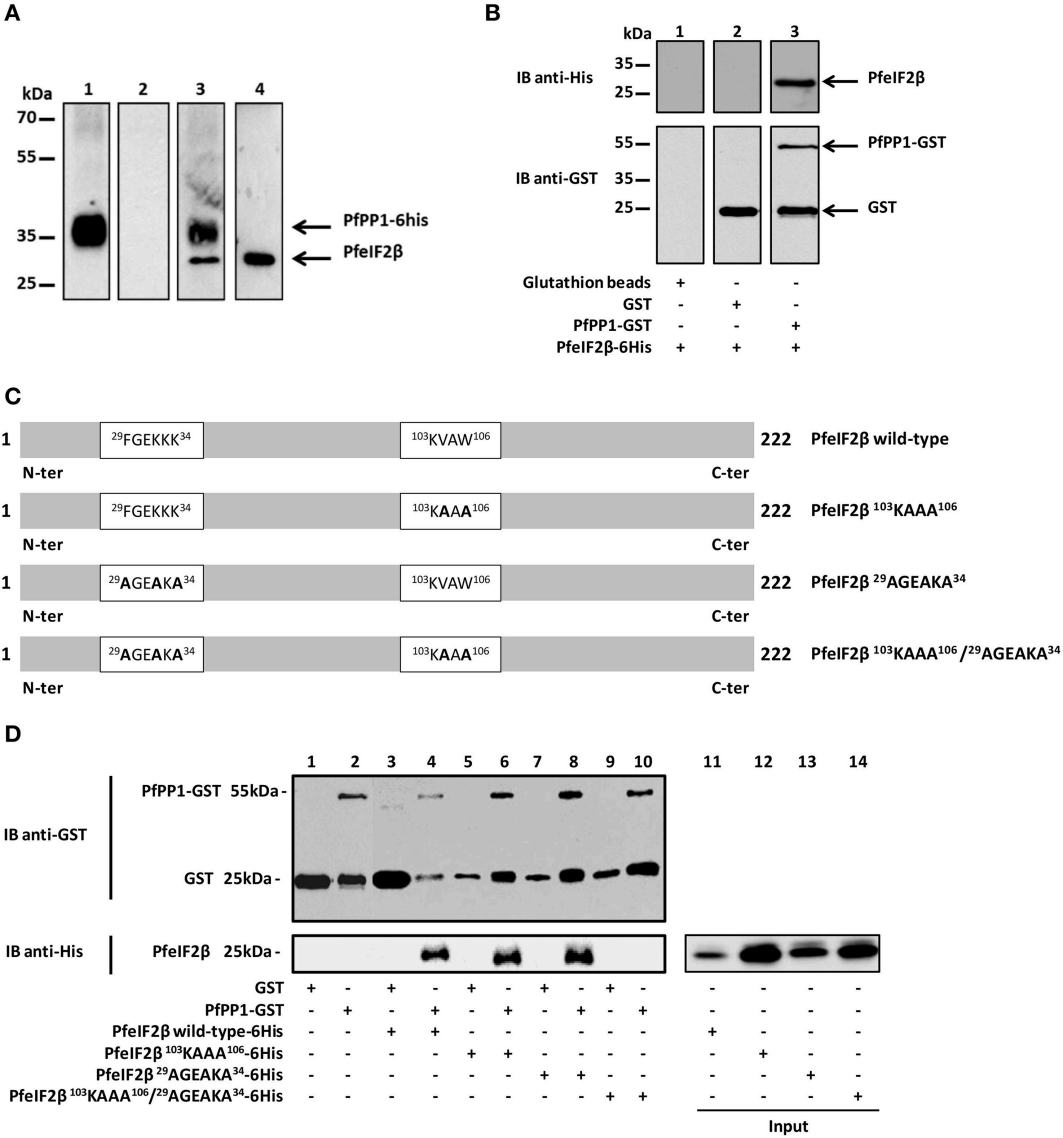
**Interaction studies of PfeIF2β with PfPP1. (A)** Binding of His-tagged PfPP1 recombinant protein to endogenous PfeIF2β expressed by *P. falciparum*. Total soluble proteins extracted from asynchronous cultures (2 mg) were pre-cleared on Ni-NTA-agarose beads before and incubated overnight with PfPP1-6His affinity Ni-NTA column. After washings, eluted proteins were separated by SDS-PAGE and blotted onto nitrocellulose. Using anti-His mAb, lane 1 confirmed the presence of His-PP1. Pre-immune serum and anti-PfeIF2β antisera were used in lanes 2 and 3 respectively. Lane 3 showed the presence of PfeIF2β. As positive control, the presence of PfeIF2β in the total parasite extracts using the PfeIF2β antisera is shown in lane 4. **(B)** Direct binding of PfeIF2β to PfPP1 by GST pull-down assays. Glutathione beads alone (lane 1) or coupled with GST alone (lane 2), or PfPP1-GST (lane 3) were incubated with 6His-tagged PfeIF2β wild-type. After washings, proteins bound to the beads were separated by SDS-PAGE and blotted onto nitrocellulose. Immunoblot analysis was performed with anti-His mAb (upper blot) and anti-GST mAb (lower blot). **(C)** Scheme representing the different versions of PfeIF2β recombinant proteins (wild-type or mutated) used in this study. **(D)** Mapping of PfeIF2β binding motifs to PfPP1. Glutathione agarose beads coupled with GST alone (lanes 1, 3, 5, 7, and 9), or GST-PfPP1 (lanes 2, 4, 6, 8, 10) were incubated with 6His-tagged PfeIF2β wild-type (lanes 3 and 4), or PfeIF2β ^103^KAAA^106^ (lanes 5 and 6), or PfeIF2β ^29^AGEAKA^34^ (lanes 7 and 8), or PfeIF2β ^103^KAAA^106^/^29^AGEAKA^34^ (lanes 9 and 10). After washings, proteins bound to the beads were separated by SDS-PAGE and blotted to nitrocellulose. Immunoblot analysis was performed with anti-GST mAb (upper blot) and with anti-His mAb (lower blot). The inputs represent 0.5 μg of each 6His-tagged protein (lanes 11, 12, 13, and 14).

The presence of two PP1 putative binding motifs in the PfeIF2β gene product led us to examine the ability of the endogenous eIF2β expressed by *P. falciparum* to bind to PfPP1. Pull-down experiments were carried out using recombinant His-PfPP1 retained on Ni-NTA agarose beads and a soluble extract of blood parasites. Eluted proteins with loading buffer were analyzed for the presence of endogenous PfeIF2β. As expected, immunoblot with anti-His mAb antibody showed the presence of His-PfPP1 (Figure 5A, lane 1). When the eluted proteins were immunoblotted with a polyclonal antibody raised against His-PfeIF2β, we observed a band at 25 kDa, corresponding to the expected size of eIF2β. An additional band was detected at 35 kDa which might correspond to His-PfPP1 as the polyclonal used could contain antibodies against the His-tagged PfeIF2β used for immunization (Figure 5A, lane 3). Although these data indicate that endogenous PfeIF2β can bind to PfPP1, it could not exclude an indirect interaction. In order to examine whether PfPP1 directly and physically binds PfeIF2β, we performed GST-pulldown experiments. Figure 5B clearly shows that GST-PfPP1 binds recombinant His-PfeIF2β (lane 3) while GST alone did not pull down PfeIF2β (lane 2).

To further clarify the molecular basis of interaction of PfeIF2β with PfPP1, we tried to identify the contribution of the putative RVxF and FxxR/KxR/K binding motifs present in PfeIF2β. To this end different versions of mutated PfeIF2β containing either single or combined mutations of putative binding motifs (Figure 5C) were used in GST-PP1 pulldown experiments. Bacterially expressed proteins were purified and incubated with GST-PfPP1 *in vitro*. As expected, wild-type PfeIF2β protein bound PfPP1 (Figure 5D, lane 4). PfeIF2β proteins with a single mutation in one binding motif (PfeIF2β ^103^KAAA^106^ or PfeIF2β ^29^AGEAKA^34^) retained their ability to bind PfPP1 (Figure 5D, lanes 6 and 8 respectively). However, PfeIF2β protein with combined mutations in the two motifs (PfeIF2β ^29^AGEAKA^34^/^103^KAAA^106^) was defective in PfPP1-binding (Figure 5D, lane 10). GST alone did not pull down wild-type PfeIF2β or any mutated protein used (Figure 5D, lanes 3, 5, 7, and 9). These data indicate that the RVxF and FxxR/KxR/K motifs are the central binding motifs of PfeIF2β to PfPP1 and that either native motif is sufficient to mediate binding.

### Induction of G2/M transition of xenopus oocytes by PfeIF2β

**Figure 6:**
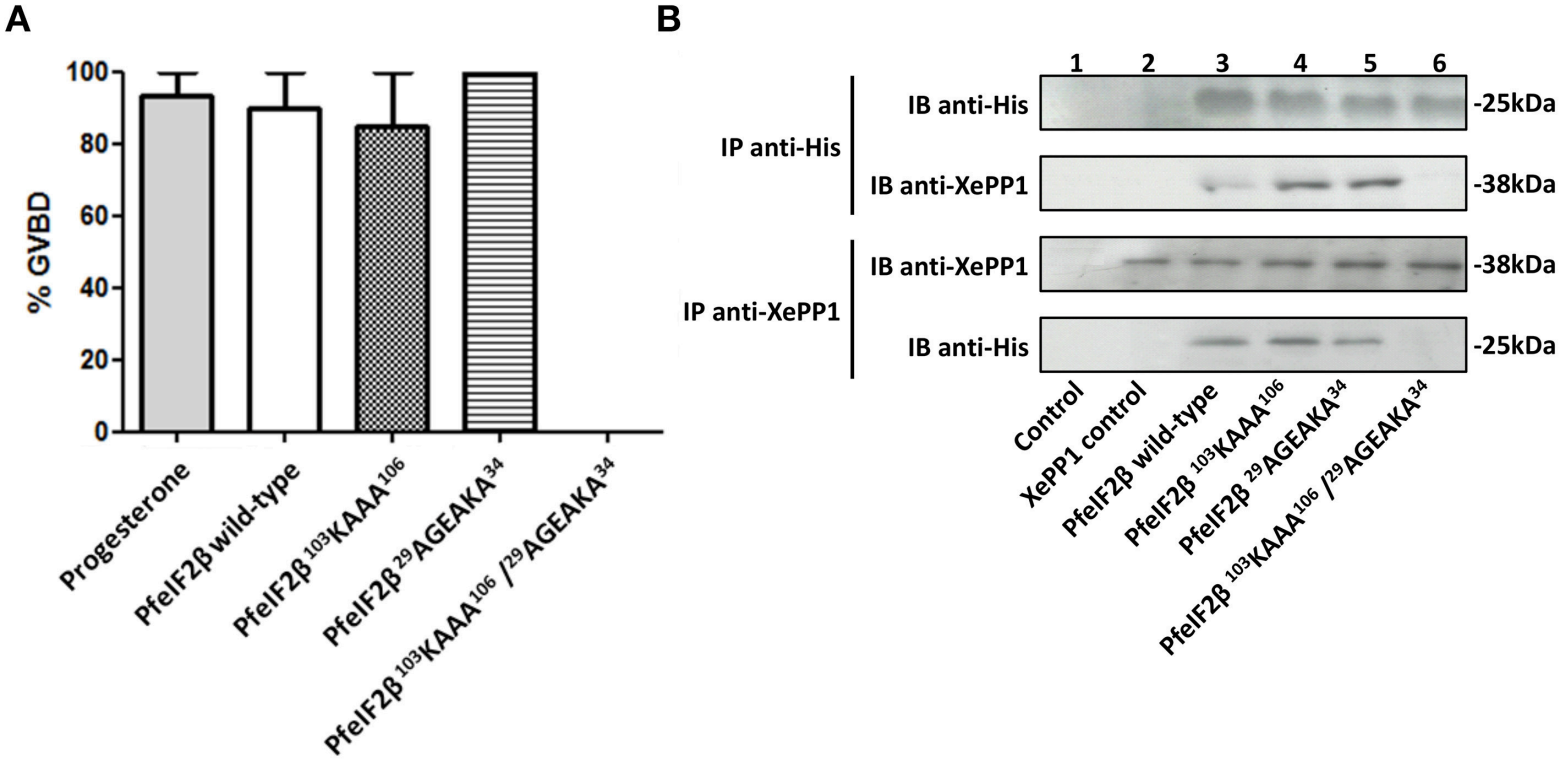
**Effect of PfeIF2β on GVBD induction in ***Xenopus*** oocytes. (A)** Induction of Germinal Vesicle Break Down (GVBD) in *Xenopus* oocytes by PfeIF2β. Each oocyte was micro-injected with 60 ng of PfeIF2β wild-type, or PfeIF2β ^103^KAAA^106^, or PfeIF2β ^29^AGEAKA^34^, or PfeIF2β ^103^KAAA^106^/^29^AGEAKA^34^ recombinant protein. Appearance of GVBD was monitored 15 h after injection. Each experiment was performed using a set of 20 oocytes. Results are presented as percentage ± SEM of four independent experiments (20 oocytes for each protein). **(B)** Binding of PfeIF2β to *Xenopus* oocytes PP1. Co-immunoprecipitation experiments with anti-His (upper blot) or anti-XePP1 (lower blot) antibodies were carried out on extracts obtained from oocytes micro-injected with wild-type, single mutated, or double mutated proteins. The anti-mouse IgG antibody was used as a control. Immunoprecipitates from oocytes were eluted, separated by SDS-PAGE and transferred to a nitrocellulose membrane. Immunoblot analysis was performed with anti-His antibodies (recognizing PfeIF2β) or anti-XePP1 antibodies.

To evaluate the impact of PfeIF2β on the activity of PfPP1 and in the absence of *Plasmodium* specific substrate to this enzyme, nonspecific substrates such as pNPP or phosphopeptide (K-R-p-T-I-R-R) were tested. Although PfPP1 was able to dephosphorylate these substrates, no effect was observed when PfeIF2β was added at different concentrations (not shown). We therefore turned our attention to the *Xenopus* oocyte model in which the micro-injection of phosphatases regulators could regulate the G2/M transition assessed by the appearance of Germinal Vesicle Break Down (GVBD) (Daher et al., 2007a; Fréville et al., 2013; Vandomme et al., 2014). In this context, we reasoned that the GVBD could serve as a surrogate marker to evaluate the functional role of the complex eIF2β-PP1. Using this model, we first showed that a micro-injection of 60 ng of recombinant PfeIF2β protein was able to induce GVBD in all micro-injected oocytes (Figure 6A). To further confirm these results, wild-type and different single or double mutated PfeIF2β proteins were used. Results depicted in Figure 6A showed that all protein versions did induce GVBD except the PfeIF2β double mutated in RVxF and FxxR/KxR/K motifs. To explain the effect of PfeIF2β in *Xenopus* oocytes, we checked whether PfeIF2β could bind to XePP1. Immunoblot analysis of eluates from either anti-His or anti-XePP1 immunoprecipitations revealed the co-immunoprecipitation of PfeIF2β and XePP1 (Figure 6B, lane 3). The single mutation of either RVxF or FxxR/KxR/K motifs did not affect the binding of PfeIF2β to XePP1 (Figure 6B, lanes 4 and 5) while the double mutation of these two motifs did abolish the binding capacity of PfeIF2β (Figure 6B, lane 6). Collectively, these data suggest that PfeIF2β rather blocked the PP1 activity in oocytes and one of the two binding motifs is sufficient for PfeIF2β to fulfill to its function.

### Reverse genetics in *P. falciparum*

**Figure 7:**
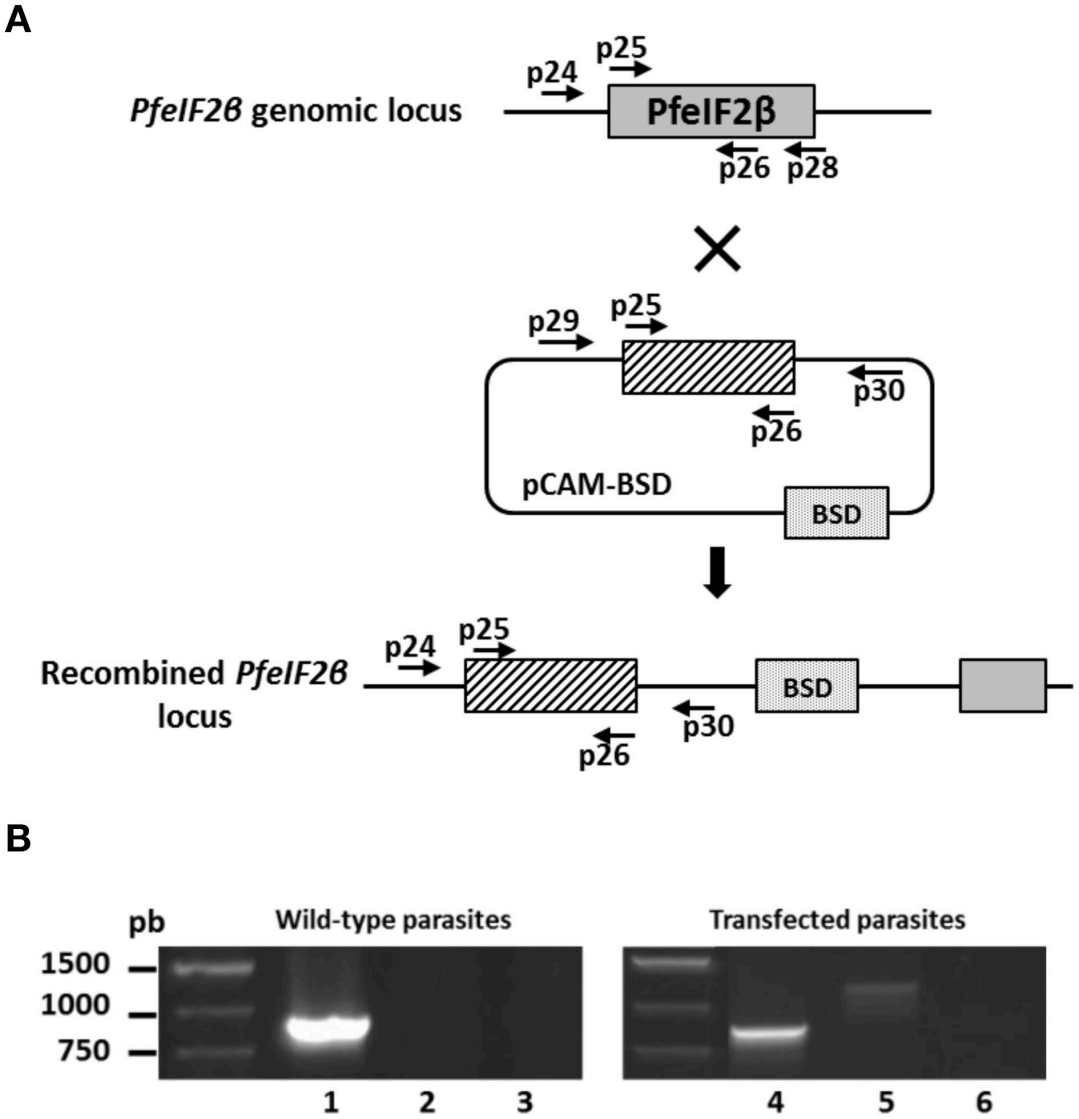
**Targeted gene disruption of the PfeIF2β locus. (A)** Gene-targeting construct for gene disruption by single homologous recombination using pCAM-BSD, and the locus resulting from integration of the Knock-Out construct. **(B)** Analysis of pCAM-BSD-PfeIF2β-transfected 3D7 cultures by PCR; lanes 1–3 correspond to DNA extracted from wild-type parasites; lanes 4–6 correspond to DNA extracted from transfected parasites. Lanes 1 and 4 represent the detection of the full-length wild-type locus (PCR with p25 and p28); lanes 2 and 5 represent the detection of episomal DNA (PCR with p29 and p30); lanes 3 and 6 represent the detection of integration of the insert (PCR with p24 and p30). The absence of a PCR product in lane 6 indicates the lack of homologous integration.

**Figure 8:**
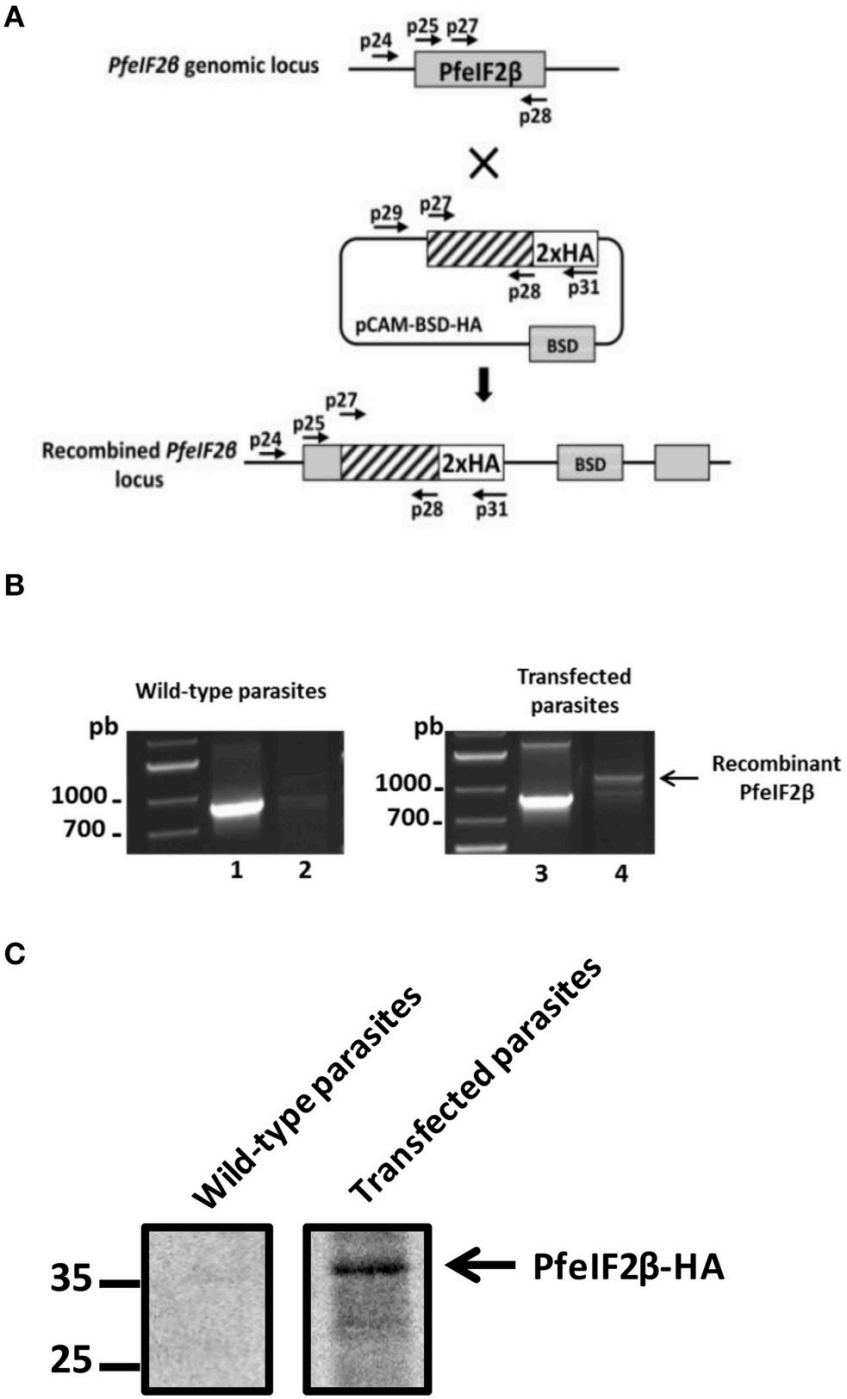
**HA-tagging of the PfeIF2β locus. (A)** Epitope tagging of PfeIF2β by Knock-In strategy. Insertion of an HA epitope tag at the C-terminus of PfeIF2β single homologous recombination. **(B)** Analysis of pCAM-BSD-HA-PfeIF2β-transfected 3D7 cultures by PCR; lanes 1–2 correspond to DNA extracted from wild type parasites; lanes 3–4 correspond to DNA extracted from transfected parasites. Lanes 1 and 3 represent the detection of the wild-type locus (PCR with p25 and p28); lanes 2 and 4 represent the detection of integration at the 5′ end of the insert (PCR with p24 and p31). The presence of a PCR product (arrow) and its sequencing confirmed the integration of a tagged *HA-PfeiF2b* gene in the locus. **(C)** Expression of HA-PfeIF2β was checked by Western-blot with anti-HA-biotin antibody after separation on SDS-PAGE. Lane 1 represents the culture of wild-type parasites and lane 2 represents the culture of transfected parasites.

To explore the role of PfeIF2β in the *P. falciparum* blood stage life cycle, silencing its expression by disrupting the gene using the pCAM vector system was attempted. Blood ring stage parasites of the 3D7 strain were transfected with a pCAM-BSD-PfeIF2β construct containing a 5′ fragment derived from the genomic *Pfeif2*β sequence and the blasticidin resistance gene (Figure 7A). From three independent transfection experiments, the analysis of genomic DNA obtained from resistant stable parasites by PCR (from 2 months up to 6 months of culture under blasticidin pressure) with specific primers (Table S1) did not detect the presence of viable knock out parasites (Figure 7B, lane 6). The wild type *Pfeif2*β gene was still amplified in genomic DNA and the plasmid remained episomal even after prolonged culture (Figure 7B, lanes 4 and 5 respectively). At this stage, it could not be excluded that the absence of viable parasites with an interrupted *Pfeif2*β gene could be attributed to the lack of accessibility of *Pfeif2*β gene locus. To examine this hypothesis, we introduced a targeted modification in the locus without loss-of-function by transfecting ring stage parasites with a plasmid containing the 3′ end of the *Pfeif2*β coding region fused to the HA sequence (Figure 8A). Using a specific primer of *Pfeif2*β derived from the upstream region of the construct *PfeIF2*β*-HA* and a primer corresponding to the HA sequence (Table S1), genotype analysis by PCR showed the correct integration of *Pfeif2*β*-HA* into the locus (Figure 8B, lane 4) and indicates the accessibility of the *Pfeif2*β locus to genetic manipulations. The integrity of the HA-tagged PfeIF2β was confirmed by immunoblot using an anti-HA monoclonal antibody and protein extracts from Knock-in parasites (Figure 8C). In order to further explore the knock in parasites, we attempted to establish stable clonal lines by limiting dilution. Surprisingly, we were unable to isolate clones expressing PfeIF2β-HA which could be attributed to the low efficiency of transfection and recombination in *P. falciparum*. Nevertheless, the above data suggest that PfeIF2β is likely essential for the development of intraerythrocytic stages of *P. falciparum*.

### Localization and subcellular fractionation of PfeIF2β

**Figure 9:**
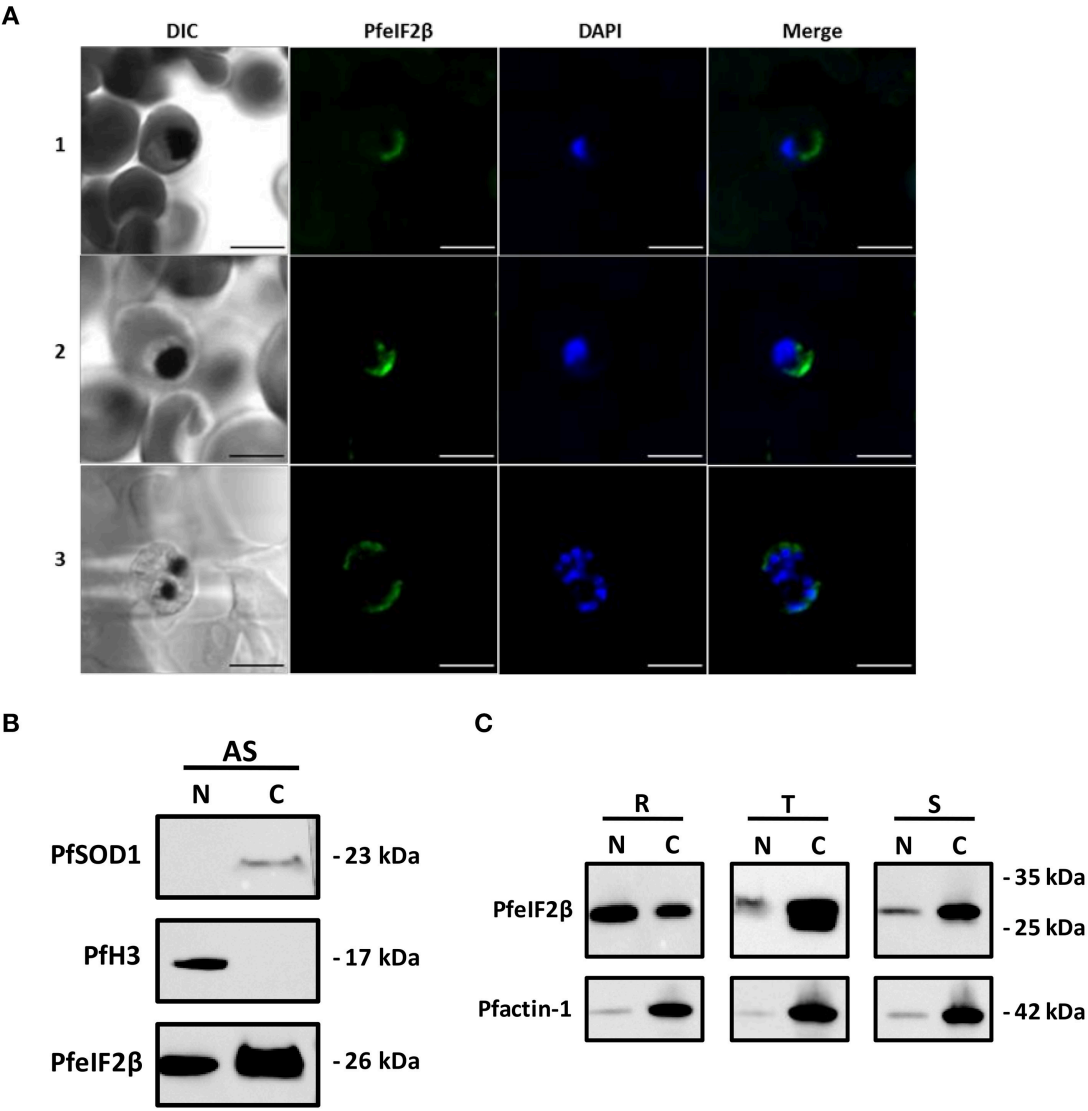
**Localization of PfeIF2β. (A)** Immunolocalization assays. Asynchronous cultures of PfeIF2β-HA tag recombinant strain of *P. falciparum* 3D7 were fixed with formalin and paraffin embedded. Sections were incubated with an anti-HA tag (biotin) antibody recognized by a streptavidine-Alexa fluor 488-labeled conjugated added with DAPI to label nuclei. Fluorescence staining was analyzed using a Zeiss LSM880 confocal microscope. The merged image of the double stained (PfeIF2β-HA tag, DAPI) and differential interference contrast (DIC) images are also presented. Immunofluorescence assays revealed a cytoplasmic localization of PfeIF2β in the trophozoite (panel 1), young schizont (panel 2), or mature schizont (panel 3) stages. Note that no staining was observed in ring-stage. No staining was observed when the primary antibody (anti-HA) was omitted (not shown). Bar = 5 μm. **(B)** Immunoblot analysis on nuclear and cytoplasm fractions from asynchronous parasites cultures. The quality control of nuclear (N) and cytoplasm (C) fractions were checked using Anti-SOD1 (upper panel) and anti-H3 antibodies (middle panel) respectively. The (lower) panel showed the presence of PfeIF2β in both fractions. **(C)** A representative Western blot assay showing the detection of PfeIF2β in cytoplasm and nuclear extracts (upper panel) from ring-stage (R), trophozoit-stage (T), and schizont-stage (S). Equal amount of nuclear and cytoplasmic proteins (10 μg) extracted from each stage were loaded. The (lower) panel showed the detection of Pf-actin 1 in the different nuclear and cytoplasmic fractions used.

In previous report, it has been demonstrated that eIF2β is exclusively present in cytoplasm of mammalian cells (Bohnsack et al., 2002). We first sought to investigate the localization of PfeIF2β in blood stage parasites by IFA. To this end, anti-HA-biotin antibody (for transfected parasites) or anti-eIF2β antisera and fixed/permeabilized thin smears of wild parental parasites on glass slides, fixed/permeabilized parasites in suspension or thin sections of fixed parasites were used. As shown in Figure 9A, only the use of anti-HA-biotin antibody combined with thin sections of fixed parasites resulted in staining of late transfected parasites (trophozoites, schizonts). The signal was mainly detectable within the parasite and seems to be restricted to the cytoplasm as no overlapping was observed with the nuclear staining. Unfortunately, all assays aiming to express GFP-tagged PfeIF2β either by episomal expression or by gene replacement failed so far. The prediction of classical nuclear localization signals in PfeIF2β (Nguyen Ba et al., 2009) incited us to further examine the parasite compartments that contain PfeIF2β using subcellular fractionation method. The use of cytoplasmic and nuclear markers confirmed the absence of protein cross contamination during fractionation (Figure 9B, upper and middle panels). Immunoblots depicted in Figure 9B (lower part) clearly confirmed the presence of PfeIF2β in the cytoplasm fraction obtained from asynchronous cultures. Intriguingly, PfeIF2β was also detectable in the nuclear fraction. The difference of data between immunoblots and IFA as to the presence of PfeIF2β in the nucleus could be attributed to a poor accessibility of the protein and/or its low abundance in this compartment. When extracts corresponding to about 10 μg of each fraction from synchronized parasite populations were tested, PfeIF2β was detected in cytoplasm and nuclear fractions obtained from ring, trophozoite, and schizont stages (Figure 9C, upper panel). The relative quantification of band intensities of PfeIF2β revealed about 2.5-fold higher in the nuclear extracts when compared to the cytoplasm fractions extracted from ring forms. However, about 11-fold and 10.5-fold increase were detected in the cytoplasm extracts when compared to nuclear extracts of trophozoites and schizonts respectively. When the anti-actin antibody was used, results showed that the actin abundance is comparable between nuclear fractions extracted from ring, trophozoite and schizont stages (Figure 9C, lower panel). Similar results were obtained with the actin detected in the cytoplasmic extracts. This supports the comparison of the level of PfeIF2β in the same compartment of each stage and further strengthens the differential distribution of PfeIF2β during the progression of blood stage parasites.

## Discussion

The coordination of PP1 regulatory networks in mammalian cells and yeast has been extensively examined (Ceulemans and Bollen, 2004) but its study is still in its early stages in *P. falciparum*. In previous studies, we have identified and characterized three PP1 regulators in *P. falciparum* for which substantial differences have been observed (protein size and functions) when compared to their counterparts in mammals. In the present study, we report the identification of PfeIF2β, a well-known component of the translational machinery, and show that it directly binds to PfPP1, although PfeIF2β is 30% shorter than its homologs. Sequence analyses clearly revealed the absence of 111 amino acids at the N-terminal region of PfeIF2β. Initial studies carried out on yeast and human eIF2β have shown that they contain three different functional regions: the N-terminal, C-terminal, and the central regions (Hashimoto et al., 2002; Gutiérrez et al., 2004). The main features of the N-terminal region of the human eIF2β (residues 1–140), lacking in *Plasmodium*, are the presence of three lysine blocks, the eIF5 binding domain (Asano et al., 1999) and two phosphorylation sites crucial for the control of protein synthesis. Indeed, mutational studies of Ser^2∕67^ revealed that these phosphorylation sites are required for its function (Llorens et al., 2006). Moreover, the overexpression of human eIF2β with its N-terminal region (residues 2–138) deleted, which is unable to bind eIF5, has been shown to be highly detrimental to cell viability (Llorens et al., 2006). These early observations with our phylogenetic analysis support the idea that the N-terminal region was acquired during evolution and is likely necessary for mammals, but it is not present in *Plasmodium*. Further structural and functional analyses revealed that the central region of eIF2β is essential for its interaction to eIF2γ subunits (Hashimoto et al., 2002), providing a platform for protein synthesis initiation (Sokabe et al., 2006; Yatime et al., 2007). Consequently, we cloned PfeIF2γ and examined its capacity to interact with PfeIF2β. Our results showed that PfeIF2β was able to bind PfeIF2γ, supporting the role of conserved regions in PfeIF2β and the tertiary structure proposed in this study (Figures 2, 3). The discovery that the PfeIF2β sequence contains 2 potential motifs for binding to PP1 has naturally raised the question of its capacity to carry out this function. PfeIF2β contains the canonical RVxF binding motif (KVAW), (also present in Amphibians and mammals) and the FxxR/KxR/K motif (FGEKKK). We have demonstrated that the endogenous eIF2β expressed by *P. falciparum* as well as the recombinant PfeIF2β protein were able to bind PfPP1. This indicates that PfeIF2β directly interacts with PfPP1 and that post-translational modifications are not a prerequisite for the binding. Furthermore, we have shown by independent experimental approaches that the putative RVxF and FxxR/KxR/K motifs present in the N-terminal region are both functional and their combined mutations completely abolished the interaction with PfPP1. Our results are in agreement with previous studies showing the involvement of the RVxF motif in human eIF2β binding. However, it has been suggested that human eIF2β harbors a second binding motif, yet to be determined, present in the C-terminal domain (between residues 187 and 314) (Wakula et al., 2006), reinforcing the idea that a different mode of interaction between PfeIF2β and PfPP1 occurs in *Plasmodium*.

To further examine the interaction of PfeIF2β-PP1 by functional assay, *Xenopus* oocytes, which are arrested in G2/M prophase I under physiological conditions, were used. In this model, the micro-injection of wild-type PfeIF2β, RVxF-mutated PfeIF2β, or FxxR/KxR/K-mutated PfeIF2β protein induced GVBD. However, the use of the double mutated version of PfeIF2β did not promote the induction of GVBD, which is in line with the incapacity of the double mutated protein to interact with XePP1 (Figure 6B). These data confirm the PfeIF2β-PP1 interaction and support the idea that PfeIF2β, in this model, is rather an inhibitor of PP1 since it has been reported that the inhibition of XePP1 by different regulators or the use of anti-PP1 antibodies did induce GVBD (micro-injection of LRR1, I2, PP1 antibodies). From these experiments, it cannot be excluded that the micro-injected PfeIF2β could be phosphorylated in oocytes, leading to an inhibitory function of PP1 in a cellular context. It is important to note that PfeIF2β is subjected to phosphorylation since phosphoproteome analyses showed the presence of phosphorylated Ser^23∕205^ and Thr^90^, raising the hypothesis that phospho-PfeIF2β could be required to impact the activity of PfPP1. Supporting this is the fact that, although recombinant PfeIF2β binds to PfPP1 *in vitro*, it did not affect the PfPP1 activity against either pNPP or small phosphopeptide. However, this should await further characterization of physiological substrates in *P. falciparum* to conclude about the role of PfeIF2β on PfPP1.

The analysis of cellular distribution of PfeIF2β revealed, as expected, its presence in the cytoplasm compartment. This localization and the capacity of PfeIF2β to bind eIF2γ and eIF5 likely support its canonical function in protein synthesis (Asano et al., 1999; Das and Maitra, 2000; Yatime et al., 2007). However, one of the most striking observations is the detection of PfeIF2β by immunoblot in the nuclear fraction under normal growth conditions. This could be an active process as PfeIF2β contains a potential nuclear localization signal. The presence of PfeIf2β in nuclear fraction could not be linked to the presence of Endoplasmic Reticulum (ER) associated organelles as the ER gradually increases during the growth of blood stage parasites (van Dooren et al., 2005). During the progression of the intraerythrocytic life cycle which coincides with an increase of the rate of translation (Foth et al., 2011), PfeIF2β shifted from the nucleus to the cytoplasm compartments. In higher eukaryotes, the shift of eIF2β localization from cytoplasm to nuclear localization can be only observed when a specific inhibitor of nuclear export was used (Bohnsack et al., 2002), suggesting a transient role of eIF2β in the nucleus. Our results combined with previous proteomic study reporting the detection of PfeIF2β in the nuclear proteome (Oehring et al., 2012) strongly suggest an unexpected steady role for this eIF2β related protein in the nucleus and mainly at the ring stage where the highest accumulation was detected. Given that we and others showed that PP1 is also nucleo-cytoplasmic, we could not rule out that PfeIF2β could interact/regulate PP1 in both compartments (Daher et al., 2006; Guttery et al., 2014). Another interesting point is that PfeIF2β can exert other functions since an earlier study reported that human eIF2β was phosphorylated by a nuclear kinase and could be a part of a protein-DNA complex (Ting et al., 1998).

Finally, in an attempt to explore the role of eIF2β in *P. falciparum* life cycle, we tried to disrupt its gene. Our studies most probably suggest an essential role for PfeIF2β in blood stage parasites, which is expected as eIF2β is required for the translation initiation complex. This is in agreement with earlier studies indicating that a deficiency in eIF2β expression in mice (eIF2S2), affects embryonic and germ cell proliferation and causes embryonic death (Heaney et al., 2009). These observations, along with the considerable difference of the gene product size of mammalian eIF2β and PfeIF2β and the divergence of the motifs/regions involved in the binding of PfeIF2β to its partners, it appears that interfering specifically on PfeIF2β functions could be conceivable and could represent an attractive approach for pharmacological intervention to control and inhibit *Plasmodium* growth.

## Author contributions

JK and CP designed the study. GT, AL, KC, AC, JV, AM, EA, and BD performed experiments. GT, AL, KC, AC, JV, AM, EA, PG, BD, AF, CP, and JK analyzed data. GT, AL, AC, JV, CP, and JK wrote the paper. All authors read, contributed feedback to, and approved the final manuscript.

### Conflict of interest statement

The authors declare that the research was conducted in the absence of any commercial or financial relationships that could be construed as a potential conflict of interest.
