# Supplementary material for: Identification of Plasmodium falciparum Translation Initiation eIF2β Subunit: Direct Interaction with Protein Phosphatase Type 1
Source: Front Microbiol. 2016 May 26;7:777. doi: 10.3389/fmicb.2016.00777 (PMC4881399; doi:10.3389/fmicb.2016.00777)
Supplement: Supplementary file 1 [file Table1.PDF]

Table S1 : List of primers used throughout this study.

Primer Number	Sequence	Orientation	Restriction site	Tagged gene	Vector	Use
**p1**	GGATCCGGAAGATAAAGTTGAAGATGCTGG	F	BamHI	6-His	pETDuet-1	PfeIF2β recombinant protein
**p2**	GGATCCATGGAAGATAAAGTTGAAGATGCTGG	F	BamHI	GST	pGEX4T3	PfeIF2β recombinant protein
**p3**	GTCGACTTAATTAGTATGCTTGGCCTTTCTTC	R	SalI	6-His/GST	pETDuet-1/pGEX4T3	PfeIF2β recombinant protein
**p4**	CAGTTATTTGATGCTGGTGAGGCGAAAGCGAAAAAGAAGAAG	F		6-His	pETDuet-1	Mutagenesis PfeIF2β ^{29}AGEAKA^{34}
**p5**	CTTCTTCTTTTTCGCTTTCGCCTCACCAGCATCAAATAACTG	R				
**p6**	CGAGTTGGATCAAAAAAAGCAGCAGCGATTAATTTTAAAG	F		6-His	pETDuet-1	Mutagenesis pfeIF2β ^{103}KAAA^{106}
**p7**	CTTTAAAATTAATCGCTGCTGCTTTTTTTGATCCAACTCG	R				
**p8**	CATCACCACAGCCAGGATCCAGCTGAGCAGAATTTAGAAACTTTAGATG	F	BamHI	6-His	pETDuet-1	PfeIF2γ recombinant protein
**p9**	CCGCAAGCTTGTCGACTCAAATTGGTTCTTGAAGCTCC	R	SalI			
**p10**	GGTTCCGCGTGGATCCGCTGAGCAGAATTTAGAAACTTTAGATG	F	BamHI	GST	pGEX4T3	PfeIF2γ recombinant protein
**p11**	GGCCGCTCGAGTCGACTCAAATTGGTTCTTGAAGCTCC	R	SalI			
**p12**	GGGCATCGATACGGGATCCTGTCGTACGTTAATATTCC	F	BamHI	HA	pGADT7	PfeIF5 RNA
**p13**	CGATTCATCTGCAGCTCGAGTTATATGGCATCTATATC	R	XhoI			
**p14**	GGATCCTGGAAGATAAAGTTGAAGATGCTGGATCAG	F	BamHI	HA	pGADT7	PfeIF2β RNA
**p15**	CTCGAGTTAATTAGTATGCTTGGCCTTTCTTCGTTC	R	XhoI			
**p16**	CATATTTATTAAACTGCAGGGATCAGCTTTTGTTGATTTAG	F	PstI		pCAM-BSD-HA	PfeIF2β KI construct
**p17**	CATATGGATAGGATCCATTAGTATGCTTGGCCTTTCTTC	R	BamHI			
**p18**	CATATTTATTAAACTGCAGATGGAAGATAAAGTTGAAGATGC	F	PstI		pCAM-BSD	PfeIF2β KO construct
**p19**	CTAGAACTAGTGGATCCGTTCACCTCTTCCTAAAGCATGG	R	BamHI			
**p24**	GAACAAAACATTGAAAAGGGCCA	F				Genotype of transfected parasites Pf
**p25**	CTGCAGATGGAAGATAAAGTTGAAGATGC	F				Genotype of transfected parasites Pf (KO)
**p26**	GGATCCGTTCACCTCTTCCTAAAGCATGG	R				
**p27**	CTGCAGGGATCAGCTTTTGTTGATTTAG	F				Genotype of transfected parasites Pf (KI)
**p28**	GGATCCATTAGTATGCTTGGCCTTTCTTC	R				
**p29**	GTAATTTATGGGATAGCGATTTTTTTTACTGTCTG	F				Detection of episome
**p30**	CCAAGCGCGCAATTAACCCTCACTAAAG	R				
**p31**	GCCATATCCAAGCTTTTAAGCATAATCTGG	R				
**p32**	GAGTATATATTTAATTTTCTCCAAAACTATAAG	F				Gene walking on cDNA 5' side
**p33**	GTCGACTTAATTAGTATGCTTGGCCTTTCTTC	R				
**p34**	ACGCGTATGGAAGATAAAGTTGAAGATGCTGG	F				
**p35**	GCAGAATTAGGAACTGAAGGATC	F				Gene walking on cDNA 3' side
**p36**	GTATATTAAAAATAAAATATTAATAAAGCGTC	R				
