# Supplementary material for: Identification of Plasmodium falciparum Translation Initiation eIF2β Subunit: Direct Interaction with Protein Phosphatase Type 1
Source: Front Microbiol. 2016 May 26;7:777. doi: 10.3389/fmicb.2016.00777 (PMC4881399; doi:10.3389/fmicb.2016.00777)
Supplement: Supplementary file 2 [file Table2.PDF]

Table S2: List of proteins and accession numbers used in phylogenetic analysis

Species	Accession number
Plasmodium falciparum	XP_001347388
Plasmodium berghei	XP_674494
Plasmodium knowlesi	XP_002258744
Plasmodium vivax	XP_001614417
Plasmodium yoelii yoelii	XP_729948
Plasmodium reichenowi	CDO64537
Plasmodium cynomologi	GAB66043
Plasmodium inui	EUD67461
Plasmodium vinckei vinckei	KEG03408
Babesia equi	AFZ79629
Babesia bigemina	CDR93997
Babesia microtis	CCF74577
Babesia bovis	BAN66020
Theileria annulata	CAI74305
Theileria orientalis	BAM40450
Theileria parva	XP_765244
Hammondia hammondi	KEP64013
Eimeria acervulina	CDI81388
Neospora caninum	CBZ54565
Cryptosporidium parvum	EAK89581
Cryptosporidium muris	EEA05106
Toxoplasma gondii	XP_002368949
Gregarina niphandrodes	EZG44924
Homo sapiens	AAH00934
Mus musculus	NP_080306
Bos taurus	NP_001015621
Gorilla gorilla	XP_004032816
Camelus ferus	XP_006179326
Xenopus laevis	NP_001090288
Schizosaccharomyces pombe	NP_593772
Nanoarcheum equitans	WP_011153313
Sulfolobus solfataricus	WP_009989461
Pyrococcus sp. NA2	YP_004424137
Pyrococcus abyssi GE5	NP_127116
Thermococcus sp. ES1	WP_042679383
Thermococcus barophilus	YP_004070280
Pyrococcus yayanasii CH1	YP_0046623547
Thermococcus gammatolerans EJ3	YP_002960391

Species	Accession number
Methanococcus vanielii SB	YP_001323817
Methanopyrus kandleri AV19	NP_614250
Staphylothermus marinus F1	YP_001041106
Pyrococcus furiosus DSM 3638	NP_578210
Arabidopsis thaliana	NP_197592
Phaseolus vulgaris	XP_007142026
Cucumis sativus	XP_004139463
Sesamum indicum	XP_011080644
Camelina sativa	XP_010493208
Vitis vinifera	XP_002265847
Populus trichocarpa	XP_002325635
Populus euphratica	XP_011021060
Brassica rapa	XP_009126490
Drosophila melanogaster	NP_524043
Ceratitis capitata	XP_004525480
Bactrocera dorsalis	XP_011201992
Anopheles darling	ETN59628
Anopheles sinensi	KFB48717
Anopheles gambiae	XP_308567
Musca domestica	XP_005181706
Nasonia vitripennis	NP_001166186
Danio rerio	NP_997840
Salmo salar	NP_001135243
Esax lucius	NP_001291068
Oncorhynchus mykiss	CDQ60780
Lepisosteus oculatus	XP_006639724
Stegastes partitus	XP_008287461
Cynoglossus semilaevis	XP_008316275
Neolamprologus brichardi	XP_006785483
Entomoeba dispar	XP_001736322
Entomoeba histolytica	XP_656368
Plasmodiophora brassicae	CEP02202
Reticulomyxa filose	ETO03611
Trypanosoma vivax	CCC48049
Trypanosoma grayi	XP_009310187
Trypanosoma brucei brucei	XP_844984
Trypanosoma brucei gambiense	XP_011773583
Trypanosoma cruzi	XP_819962
