# Supplementary material for: Identification of Plasmodium falciparum Translation Initiation eIF2β Subunit: Direct Interaction with Protein Phosphatase Type 1
Source: Front Microbiol. 2016 May 26;7:777. doi: 10.3389/fmicb.2016.00777 (PMC4881399; doi:10.3389/fmicb.2016.00777)
Supplement: Supplementary file 3 [file Image1.PDF]

5’GAACAAAACATTGAAAAGGGCCATATAAATATATATTATACATATATATTATAT
TATTACGACAAATTAATAAAAGAGTATATATTTAATTTTCTCCAAAACTATAAGA
TTATTATATTAATTTAAATATAATTATTTATAAATTTTTGATAAGATAAAT**ATG**GA
AGATAAAGTTGAAGATGCTGGATCAGCTTTTGTTGATTTAGATAAAATAGTAAAT
GATGATTCGAAACAGTTATTTGATTTTGGTGAGAAGAAAAAGAAAAAGAAGAAG
AAAGAGGTTGTAGAAAAGGTAGAAGAGATTATTATAGATGGTACAGGGAAAGTT
TTTGAAAGAGGAGCTGTATATCCATATGATGAATTATTACATCGAATTCAAGATT
TAATTAATAAACATAACATAGATTTATGTATATCTAAAAAATATACTATTAAACC
CCCACAAGTAGTTCGAGTTGGATCAAAAAAAGTAGCATGGATTAATTTTAAAGAT
ATATGTACTATTATGAATAGAAATGAAGAACATGTTTTCCATTTTGTTTTAGCAGA
ATTAGGAACTGAAGGATCAATAGCAGGAGAAGGACAATTAGTTTTAAAGGGAAA
GTATGGACCTAAACATATTGAAGCACTCTTAAGAAAATATATTACTGAATATGTA
ACTTGCCAAATGTGTAAAAGTCCTAATACAACTATGGAAAAGGATAGTAGGACA
AGACTTTTTCATCAGCACTGTAATGCTTGTGGAGCAAAAAGgttagtcctatataaaaaaaaata
aatatatatgtataaatgtatatatgtatatatatatgtatatatatatgtatatatatatgtatataatatatatttatttatatatatttaataattttcat
atttctatatatttgcttctatatatatatatatatatatatatatatttttttttttttttttttttccagATCTGTTACAACCATTAA
GAGTGGTTTCCATGCTTTAGGAAGAGGTGAACGAAGAAAGGCCAAGCATACTAA
T**TAA**TGTTAAAATATATAAAAATTAAAAAATATATAAAATTTTAAAAATATAAAA
AAAGACGCTTTATTAATATTTTATTTTTAATATACTACTACCATAAGAAATATATA
TATATATATATATATAAATATATTTCTATATTAATAAATTCTTTTTTGTTTTTTTTTT
TTTTTTTTTTTTAAAAATATTCTTGATACTTTAAGTTTGTATAAAATTATATATTA3’

Supplementary Figure 1: Nucleotide sequence of PfeIF2β. cDNA was obtained by RT-PCR using different primers to confirm the start and the stop codons of PfeIF2β sequence. The 5’ and 3’ non-coding sequences are underlined. ATG and STOP codons are bolded.
