# Supplementary material for: Identification of Plasmodium falciparum Translation Initiation eIF2β Subunit: Direct Interaction with Protein Phosphatase Type 1
Source: Front Microbiol. 2016 May 26;7:777. doi: 10.3389/fmicb.2016.00777 (PMC4881399; doi:10.3389/fmicb.2016.00777)
Supplement: Supplementary file 4 [file Image2.PDF]

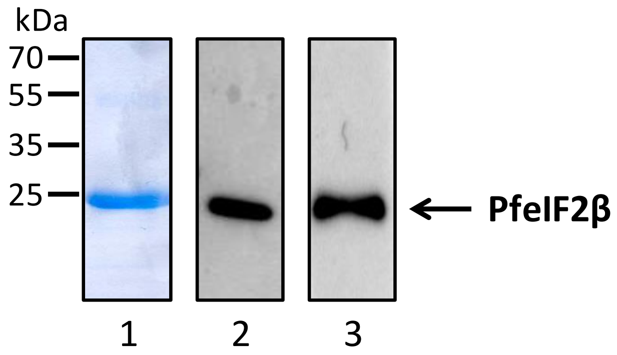

Supplementary Figure 2: Expression of PfeIF2β protein. Lane 1 represents the stained with Coomassie Blue, lane 2 represents the immunoblot of purified PfeIF2β-6His revealed with anti-His mAb and lane 3 the immunoblot using an antisera raised against PfeIF2β. A single band at ~ 25kDa is observed, corresponding to the expected size of the recombinant protein. The identity of the purified recombinant PfeIF2β was further confirmed by MALDI-TOF mass spectrometry.
