# Supplementary material for: Identification of Plasmodium falciparum Translation Initiation eIF2β Subunit: Direct Interaction with Protein Phosphatase Type 1
Source: Front Microbiol. 2016 May 26;7:777. doi: 10.3389/fmicb.2016.00777 (PMC4881399; doi:10.3389/fmicb.2016.00777)
Supplement: Supplementary file 5 [file Image3.PDF]

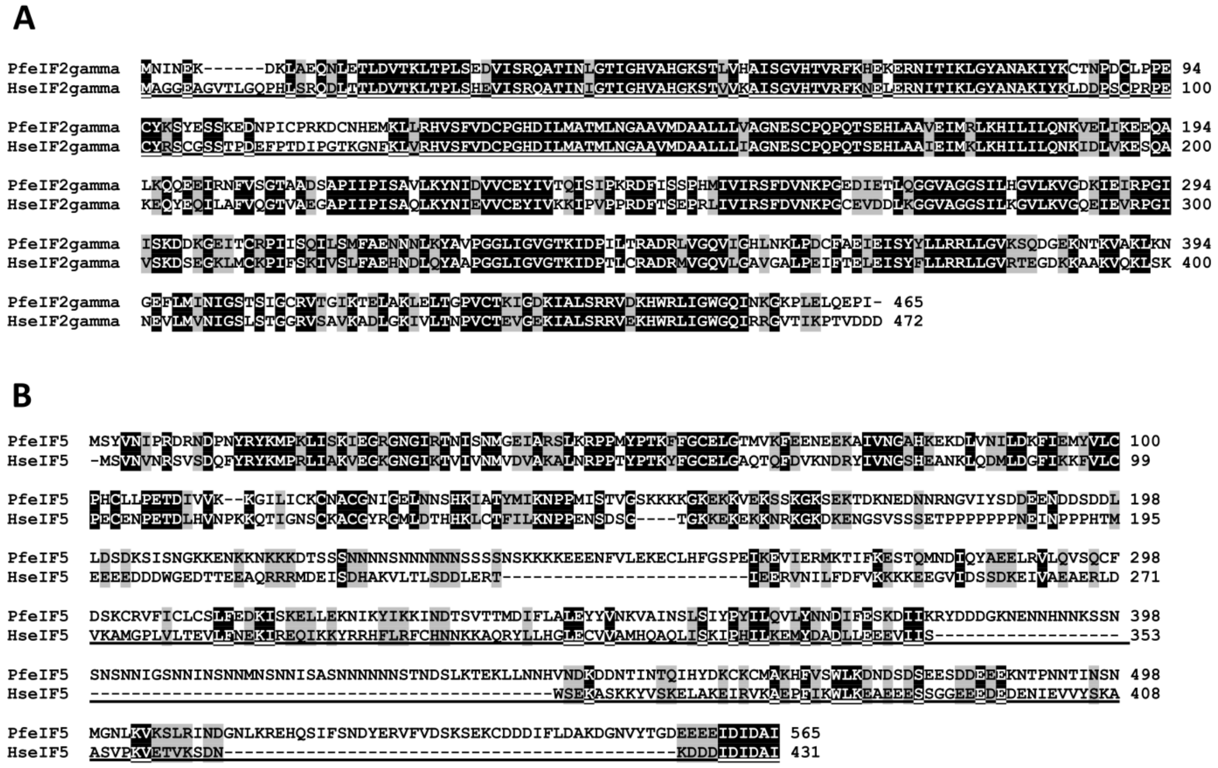

Supplementary Figure 3: Comparison between the deduced amino acid sequence of *P. falciparum* and Human eIF2γ and eIF5. PfeIF2γ (PF3D7_1410600) **(A)** and PfeIF5 (PF3D7_1206700) **(B)** were aligned with the human eIF2γ (Genbank accession number NP_00146.1) and eIF5 (Gen bank accession number NP_892116.2) orthologues respectively, using the ClustalW algorithm. The identical and semi-conserved amino acids are highlighted in black and grey respectively. Amino acids underlined in grey represent the part that interacts with eIF2β.
